# Adrenergic signalling to astrocytes in anterior cingulate cortex contributes to pain-related aversive memory in rats

**DOI:** 10.1038/s42003-022-04405-6

**Published:** 2023-01-05

**Authors:** Zafar Iqbal, Zhuogui Lei, Aruna S. Ramkrishnan, Shu Liu, Mahadi Hasan, Mastura Akter, Yuk Yan Lam, Ying Li

**Affiliations:** 1 Department of Neuroscience, College of Veterinary Medicine and Life Sciences, City University of Hong Kong, Kowloon, Hong Kong; 2 Department of Biomedical Sciences, College of Veterinary Medicine and Life Sciences, City University of Hong Kong, Kowloon, Hong Kong; 3 Centre for Regenerative Medicine and Health, Hong Kong Institute of Science & Innovation, Chinese Academy of Sciences, Hong Kong SAR, China; 4 Centre for Biosystems, Neuroscience, and Nanotechnology, City University of Hong Kong, Kowloon, Hong Kong

**Keywords:** Astrocyte, Learning and memory

## Abstract

Pain contains both sensory and affective dimensions. We identify the role of norepinephrine in colorectal distention (sub-threshold for acute pain) induced conditioned place avoidance and plasticity gene expression in the anterior cingulate cortex (ACC). Activating locus coeruleus (LC)-projecting ACC neurons facilitates pain-evoked aversive consolidation and memory, while inhibiting LC-projecting ACC neurons reversibly blocks it. Optogenetic activation of ACC astrocytes facilitates aversive behaviour. ACC astrocytic Gi manipulation suppressed aversive behaviour and early plasticity gene expression induced by opto-activation of LC neurons projecting to ACC. Evidences for the critical role of β2AR in ACC astrocytes were provided using AAV encoding β2AR miRNAi to knockdown β2AR in astrocytes. In contrast, opto-activation of ACC astrocytic β2ARs promotes aversion memory. Our findings suggest that projection-specific adrenergic astrocytic signalling in ACC is integral to system-wide neuromodulation in response to visceral stimuli, and plays a key role in mediating pain-related aversion consolidation and memory formation.

## Abstract (web summary)

Chemogenetic and optogenetic profiling in a rat model of acute pain-related unpleasantness suggests that colorectal distention-induced place aversion is mediated by locus coeruleus noradrenergic signalling to astrocytes in anterior cingulate cortex.

## Introduction

Pain is a conscious subjective experience that is most often invoked by nociceptive stimulation, activation of nociceptors and nociceptive pathways undoubtedly cause pain. On the other hand, abundant evidence indicates that nociceptors can be active in the absence of pain perception^1^. Pain involves both sensory and affective elements. Previous electrophysiological studies have identified colorectal distention (CRD)-responsive neurons in the ACC^2–4^. We determined that acute splanchnicectomy combined with pelvic nerve sectioning completely eliminated the ACC neuronal responses evoked by CRD^2^ suggesting the peripheral afferent inputs from CRD are transmitted through the pelvic and splanchnic nerves to the ACC evoking ACC neuronal responses to CRD^2^. Using a chronic visceral hypersensitive rat model sensitized to chicken egg albumin, we have previously shown that colonic anaphylaxis increases anterior cingulate cortex (ACC) sensitization. Performing colorectal distention (CRD) with noxious distention pressure induced visceromotor reflex^5^ (a pseudo-affective reflex) as pain perception, allodynia and hyperalgesia were characterized in viscerally hypersensitive rats^2–4,6^. Alterations of synaptic plasticity in medial thalamus-ACC synapses have been reported in viscerally hypersensitive rats^3^.

The affective dimension of pain is made up of feelings of unpleasantness. The ACC has a crucial role in the affective-aversive experience of pain^7–11^. The mechanisms for enhancement of aversion in the chronic pain state have been widely investigated using noxious stimulation. Previous studies using animal models with chronic pain have shown the recruitment of acute pain processing during retrieval of pain-conditioned passive avoidance^12,13^.

Human brain imaging studies and rodent studies in aversive behavioural learning have provided ample evidence that pain perception is distinct from nociception^1^. With time and previous emotional learning, nociceptive activation manifests into pain itself^14^. While pain is comprised of sensory and affective elements, animal pain models are notably lacking in a behavioural index to evaluate the affective component of pain. Using a rodent pain-related assay that combines colorectal distension (CRD magnitude ≤35 mmHg) with the conditioning place avoidance (CPA), we measured a learned behaviour that directly reflects the affective component of pain evoked by visceral stimulation and develops considerable aversion associative learning and memory^15,16^. In this study, we use CRD with magnitude ≤ 35 mmHg, a sub-threshold for pain perception, as a nociceptive stimulation, combined with CPA paradigm to demonstrate that when CRD was paired with a distinct environmental context, rats spent significantly less time in this distinct environment on the post-conditioning test days as compared with the pre-conditioning day, indicating that rats subjected to visceral nociceptive stimulation experienced significant aversion which can support associative learning and memory^15,16^.

The LC plays a substantial role in the modulation of alertness and acuity. The LC has long-ranging and highly branched unmyelinated projections spanning the cortex, cerebellum, and subcortical nuclei; it is involved in the regulation of complex cognitive processes such as the consolidation of long-term memory^17,18^. Pain memories that are formed in a state of arousal and heightened emotion are remembered long-term. The mechanism through which adrenergic receptors mediate pain aversive long-term memory consolidation remains unclear. In the central nervous system, norepinephrine acts via α- and β-adrenergic receptors, of which β2-adrenergic receptors (β2ARs) are of particular importance. β2ARs are expressed mainly in astrocytes^19^, while β1ARs are found at the synaptic junctions of neurons^20^. The functioning of prefrontal cortex is heavily sensitive to LC-derived norepinephrine, and can be significantly altered by low levels of norepinephrine^21^. We hypothesize that visceral nociceptive stimulation induces an aroused state in which a noxious stimulus activates LC neurons to release neuromodulators in the brain which then rapidly alters LC-ACC network communication. This brain network processing is necessary for encoding, consolidation and retrieval of a long-term post nociceptive stimulation-induced aversive memory component compared to fast-acting acute pain sensation.

Astrocytes are a glial cell type that can sense and modify synaptic activity at surrounding synapses by releasing active substances^22^. Several studies have demonstrated that astrocytes are capable of releasing major neurotransmitters: glutamate, ATP, GABA, and glycine^23–28^ and subsequently affect behaviourally relevant responses^29^. Astrocytes support neuronal functions by providing neuronal energy substrates such as lactate by means of their astrocytic processes^30–32^. Noradrenergic receptors are expressed in astrocytes. A previous study showed that release of norepinephrine from LC projections enables highly coordinated astrocyte Ca^2+^ signalling in awake behaving mice^33^. Extensive astrocytic Ca^2+^ signalling has been observed in monkey dorsolateral prefrontal cortex after direct stimulation of the LC^17^, suggesting that norepinephrine contributes to the production of global astrocytic signals^34^.

βARs are thought to contribute to memory functions through exerting effects on neurons^20,35^. In the central nervous system, β2ARs are expressed mainly in astrocytes^19,36,37^, alluding to the important role of β2ARs in astrocyte – neuronal interactions in cognitive functions.

The incorporation of chemogenetic and optogenetic techniques in neuroscience research allows real-time, reversible manipulation of specific populations of cells^30,38^. In this study, we reveal that optogenetic activation of LC neurons projecting to ACC facilitates long-term visceral pain aversive memory and induction of learning-dependent c-Fos expression. These effects were suppressed by administration of βARs antagonist propranolol. In contrast, real-time silencing of LC neurons blocks aversive memory retrieval after initial CPA learning.

We postulate that if adrenergic astrocytic signalling in the ACC plays a fundamental role in modulating pain-related aversion, then chemogenetic manipulation of ACC astrocytes could have a major effect on amentia. We expressed the Gi-coupled designer receptor hM4Di in astrocytes and discovered that astrocytic Gi activation during learning and memory recall diminished CPA responses, and further suppressed aversive behaviour induced by optogenetic activation of LC neurons projecting to ACC. Moreover, ACC astrocytic Gi activation suppressed ACC plasticity gene expression in behaving animals. These findings point to the importance of astrocytes in aversive learning and memory processes, and its action via surrounding neurons. Using AAV encoding β2AR miRNAi with astrocytic promoter-glial fibrillary acidic protein (GFAP) to knockdown β2AR in astrocytes, we clarified the critical role of ACC astrocytic β2AR in modulating pain aversion. We show that enhanced pain aversive memory resulting from optogenetic activation of ACC astrocytic β2AR. Collectively, these observations support the idea that coordinated adrenergic astrocytic signalling is integral to system-wide neuromodulation and suggest that norepinephrine-astrocytic signalling in ACC plays a key role in modulating visceral nociceptive stimulation-evoked aversion consolidation and memory.

Chronic visceral pain is a key factor in the pathophysiology of irritable bowel syndrome (IBS)^39^. In clinical settings, persistent pain is often crippling, causing considerable suffering and anxiety in those who suffer from it. These findings provide insights into treatment of pain-related emotional aversions.

## Results

### Selective ablation of LC noradrenergic neurons disrupts visceral noxious aversive memory formation

**Fig. 1:**
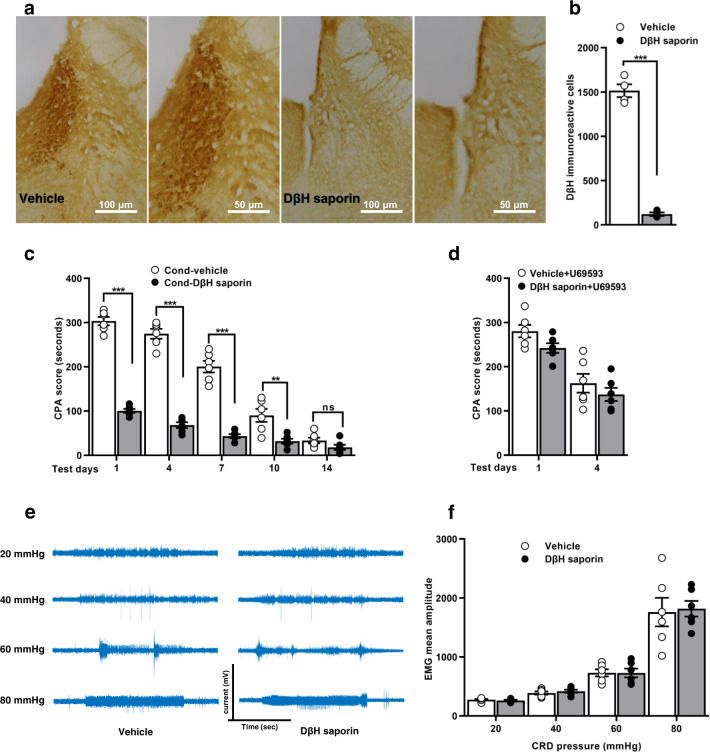
Immunotoxic ablation of locus coeruleus noradrenergic neurons impairs visceral noxious aversive memory but had no effect on acute visceral pain behaviours. **a** Representative images showing cell population of LC noradrenergic neurons in vehicle and DβH saporin injected rats. Scale bar: 100, 50 µm. **b** Quantification of DβH immunoreactivity (*n* = 4/group with 3 sections from each rat; *t*_6_ = 18.65, ***p < 0.0001, unpaired t-test). **c** CPA score in the vehicle and DβH saporin rats on test days (*n* = 6/group; ***p < 0.0001, **p < 0.001, ^ns^*p* = 0.7149, two-way ANOVA with Bonferroni test). **d** CPA score to U-69593, induced CPA in vehicle and DβH saporin rats (*n* = 6/group; *p* = 0.6881, two-way ANOVA). **e** Representative visceromotor response (VMR) recordings to graded pressure (20, 40, 60, and 80 mmHg) of colorectal distension (CRD). **f** The mean amplitude of electromyography (EMG) in response to graded CRD pressure in the vehicle and DβH saporin rats (*n* = 6/group; F_(3, 40)_ = 0.04675, *p* = 0.9864, two-way ANOVA). All results are expressed as mean ± SEM. ns = non-significant, *p* > 0.05.

We tested whether LC noradrenergic neurons contribute to aversive learning memory. The anti-DβH saporin selectively destroyed the LC noradrenergic neurons with > 93% loss of DβH immunoreactivity compared with vehicle rats (Fig. 1a, b and Supplementary Data 1). On test days, depletion of noradrenergic neurons induced a significant decrease in the CPA score (Fig. 1c; Supplementary Table 1 and Supplementary Data 1).

Next, we demonstrated that noradrenergic neurons mediate the aversion of visceral nociception, or they are associated with aversiveness in general. We examined the effects of ablation of noradrenergic neurons by an aversive but non-noxious activating stimulus, U69593, a selective κ-opioid receptor agonist. On test days, no significant difference was detected in the CPA score (Fig. 1d; Supplementary Table 1 and Supplementary Data 1). The depletion of noradrenergic neurons failed to change the aversive responses to U69593 induced CPA. The visceromotor response (VMR) to the graded pressures of colorectal distention (CRD) was performed to measure the effects of noradrenergic neuronal loss on visceral pain sensation. The LC noradrenergic lesion did not change the CRD induced VMR (Fig. 1e, f and Supplementary Data 1).

### Optogenetic silencing of LC neurons projecting to ACC impairs aversive learning and memory formation

**Fig. 2:**
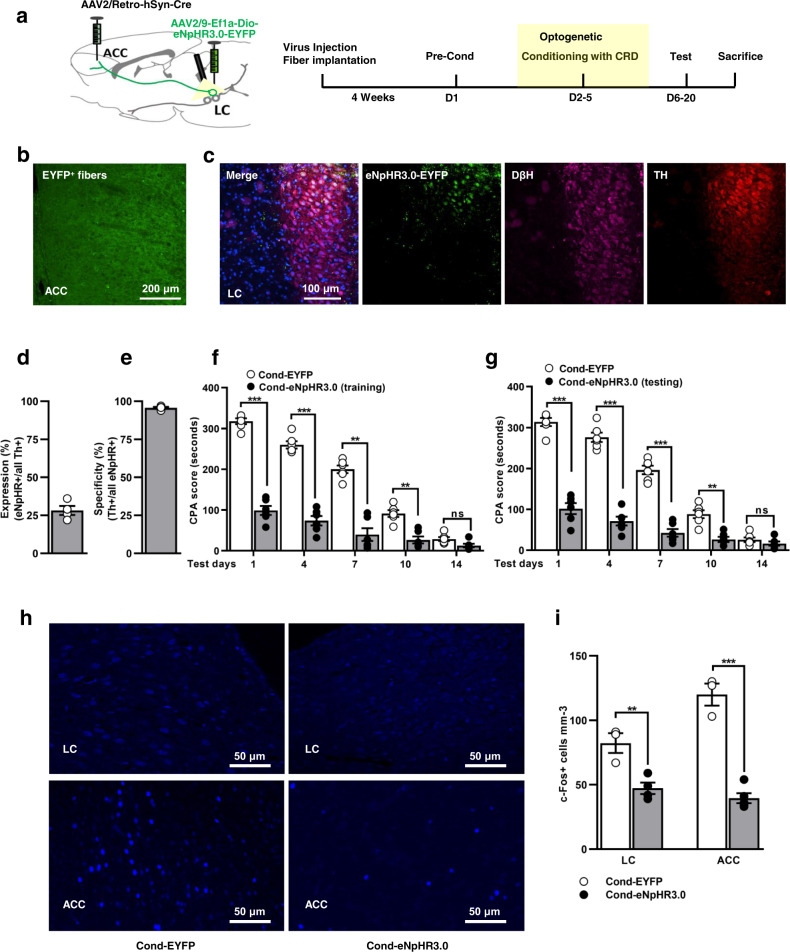
Optogenetic silencing prevents the recruitment of the locus coeruleus neurons projecting to ACC. **a** Schematic protocol depicts double viral injections of AAV2/retro-Cre and Dio-eNpHR3.0-EYFP into ACC and LC respectively, and experimental timeline for CPA behaviour. **b** Viral expression of eNPHR3.0 in LC neurons projecting to ACC. Scale bar: 200 µm. **c** Representative images of eNpHR3.0 colocalized with Th^+^ and DβH^+^ neurons in LC region. Scale bar: 100 µm. % transfection efficiency (**d**) and specificity (**e**) of eNpHR3.0 with Th^+^ neurons in LC (*n* = 4, 3 sections from each rat). **f**, **g** CPA score in EYFP and eNpHR3.0 rats when optogenetic silencing was performed during training (**f**) or before testing days (**g**; yellow light pulse 15 ms, 20 Hz frequency, 3 min ON and 3 min OFF; *n* = 6/group; ***p < 0.0001, **p < 0.001, ^ns^*p* > 0.9999, two-way ANOVA with Bonferroni test). **h** Representative images of c-Fos expression in EYFP and eNpHR3.0 rats. Scale bar: 50 µm. **i** Quantification of c-Fos^+^ cells in the LC and ACC region after optogenetic inhibition (*n* = 3–5 rats/group, 3 sections from each animal; **p < 0.0083; *t*_5_ = 4.224 (LC); ***p < 0.0001, *t*_6_ = 9.946 (ACC), unpaired t-test). Results are presented as mean ± SEM. ns = non-significant, *p* > 0.05.

The permanent loss of LC noradrenergic neurons and norepinephrine synthesis shifts the brain to start using other promoting systems of the brain regions such as those involved in food intake^40^. Therefore, this prompted us to use a real-time optogenetic tool to silence the LC neurons. We hypothesized that silencing of LC neurons projecting to ACC could modify the aversive learning memory formation. To test the hypothesis, we injected an AAV2/retro-Cre into ACC and a Cre-dependent Dio-eNpHR3.0-EYFP into LC (Fig. 2a). Stereotactic injection of double viral vectors induced the expression of eNpHR3.0-EYFP in LC neurons projecting to ACC (Fig. 2b, c and Supplementary Fig. 1a). The optogenetic actuator eNpHR3.0 with retrograde Cre-dependent construct did not express in the hippocampus, amygdala and thalamus region of the brain (Supplementary Fig. 4d). Notably, eNpHR3.0 is expressed in noradrenergic neurons with transfection efficiency > 28% and specificity > 95% (Fig. 2d, e and Supplementary Data 1). On test days, optogenetic inhibition of LC neurons, during training or before testing days, dramatically reduced the CPA score (Fig. 2a, f, g, Supplementary Table 2 and Supplementary Data 1). In contrast, optical inhibition of noradrenergic neurons in the absence of CRD has no effect on CPA score (Supplementary Fig. 3a, b and Supplementary Data 1).

To gain insight into changes in the LC neurons projecting to ACC and within ACC itself, we detected the presence of c-Fos (an indirect marker of neuronal activation). Brains were collected 90 min following light delivery to the LC neurons. We observed that optogenetic inhibition of LC neurons projecting to ACC during training significantly suppressed the c-Fos expression in LC and ACC regions (Fig. 2h, i and Supplementary Data 1). Additionally, in a separate group of rats, we found that optical inhibition of ACC projecting LC neurons before testing day 1 significantly downregulated the c-Fos expression in the LC and ACC region respectively (*p* = 0.0011, F_(2, 9)_ = 16.09 (LC); *p* = 0.0004, F_(2, 9)_ = 21.73 (ACC); Supplementary Fig. 4a–c and Supplementary Data 1). These results suggested that photoinhibition of LC neurons blocked the LC to ACC bottom-up communication during memory acquisition and expression.

### βARs antagonist propranolol and selective β2ARs antagonist ICI118,551 into ACC disrupts aversive memory and learning-dependent plasticity changes

**Fig. 3:**
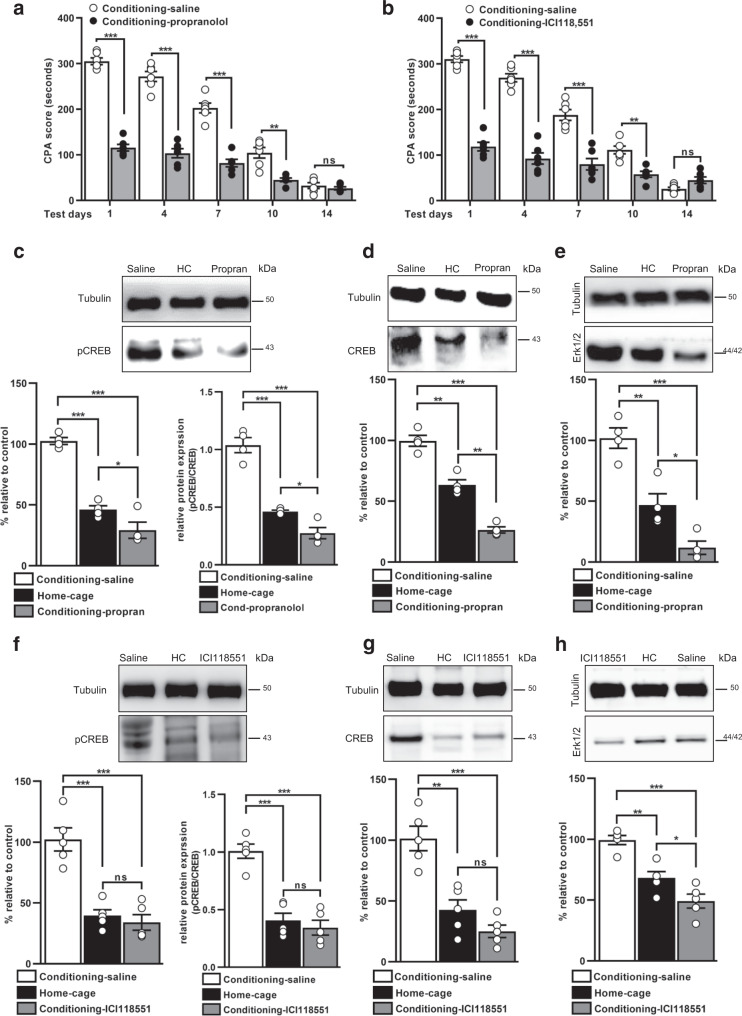
Bilateral infusion of βARs antagonist and selective β2ARs antagonist into ACC disrupts aversive memory and memory-related plasticity changes. **a** CPA score in saline and propranolol injected rats on testing days (*n* = 6/group; ***p < 0.0001, **p < 0.001, ^ns^*p* = 0.9918, two-way ANOVA with Bonferroni test. **b** CPA score recorded on test days in saline and ICI 118,551 administered rats (*n* = 6/group; ***p < 0.0001, **p < 0.001, ^ns^*p* = 0.5658, two-way ANOVA with Bonferroni test). Representative images and western blot analysis showing the expression level of pCREB (**c**), pCREB/CREB (**c**), CREB (**d**), and Erk1/2 (**e**) in the saline, home cage, and propranolol administered rats (*n* = 4/group; ***p < 0.0001, **p < 0.001, *p < 0.01, one-way ANOVA with Tukey’s test). Representative blots and western blot quantification for pCREB (**f**), pCREB/CREB (**f**), CREB (**g**), and Erk1/2 (**h**) level in saline, home cage, and ICI118,551 injected rats (*n* = 5/group; ***p < 0.0001, **p < 0.001, *p < 0.01, ^ns^*p* = 0.8424 (pCREB), ^ns^*p* = 0.7630 (pCREB/CREB), ^ns^*p* = 0.3119 (CREB), one-way ANOVA with Tukey’s test). Results are presented as protein percentage of control sample mean values (100%). Protein values are normalized to those of tubulin. All results are expressed as mean ± SEM. ns = non-significant, *p* > 0.05. Propran = propranolol.

To test whether  noradrenergic signalling plays an essential role in aversive memory formation, we infused βARs antagonist propranolol into ACC 15 min before conditioning. As expected, on testing days, propranolol had a significant decrease in CPA score (Fig. 3a, Supplementary Table 3, and Supplementary Data 1).

Next, we used a directed pharmacological approach to dissociate and manipulate the receptor specific functions of β2ARs in aversive memory. We injected bilateral injections of selective β2ARs antagonists ICI118,551 into ACC 15 min before conditioning. On test days, ICI118,551 robustly reduced the CPA score (Fig. 3b, Supplementary Table 3, and Supplementary Data 1). Further, we tested the effects of propranolol and ICI118,551 on the neuronal plasticity genes required for learning and memory formation. Quantitative western blot analyses indicated that propranolol administered rats had a significant decrease in the expression level of pCREB (*p* < 0.0001, F_(2, 9)_ = 70.32), pCREB/CREB (*p* < 0.0001, F_(2, 9)_ = 70.40), CREB (*p* < 0.0001, F_(2, 9)_ = 90.07), and Erk1/2 (*p* < 0.0001, F_(2, 9)_ = 33.36; Fig. 3c–e and Supplementary Data 1). In a separate group of rats, the quantitative western blot analysis showed a significant decrease in protein expression of pCREB (*p* < 0.0001, F_(2, 12)_ = 28.09), pCREB/CREB (*p* < 0.0001, F_(3, 12)_ = 34.08), CREB (*p* < 0.0001, F_(2, 12)_ = 24.36), and Erk1/2 (*p* < 0.0001, F_(2, 12)_ = 25.96), in ICI118,551 injected rats compared to saline group (Fig. 3f–h and Supplementary Data 1). Together, data shows that βARs receptor signalling plays a critical role in aversive memory as injections of βARs and β2ARs antagonists caused the downregulation of memory-related plasticity changes.

### Optogenetic stimulation of LC neurons that project to ACC enhances aversive memory, which is disrupted by propranolol

**Fig. 4:**
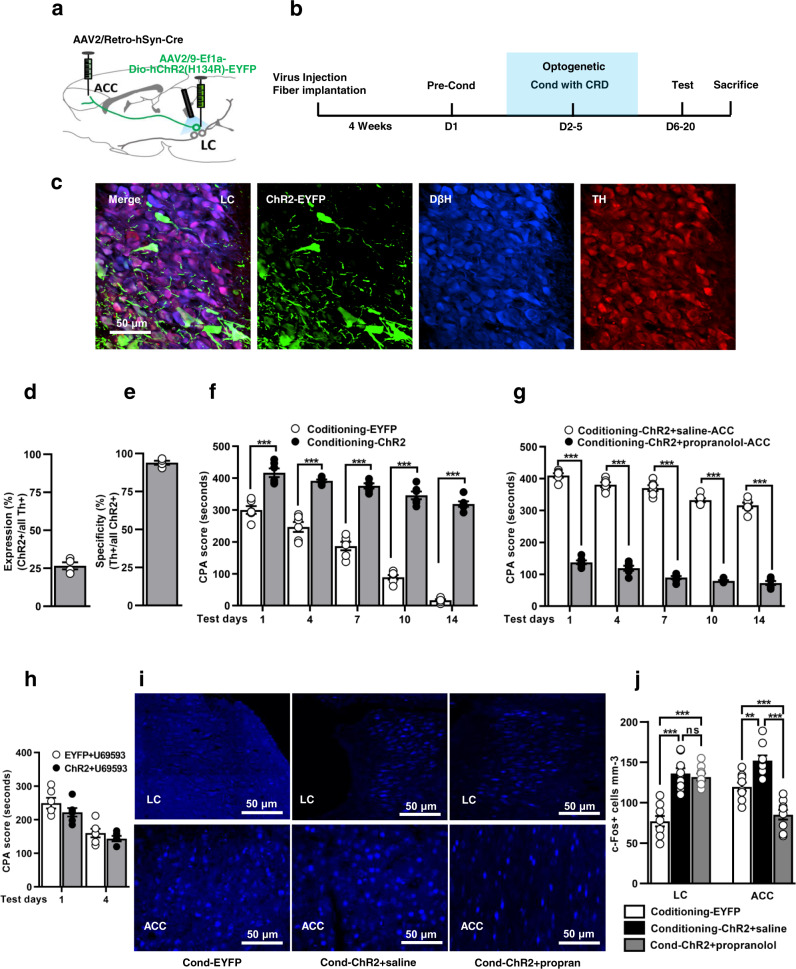
Opto-stimulation of LC neurons projecting to ACC enhances aversive memory, which is disrupted by propranolol. **a** Schematic protocol of viral injections: AAV2/retro-hSyn-Cre and AAV2/9-Dio-ChR2-EYFP. **b** Experimental timeline showing CPA protocol. **c** Representative images of ChR2^+^ cells expression in the LC noradrenergic neurons. Scale bar: 50 µm. % transfection efficiency (**d**) and specificity (**e**) of ChR2 with Th and DβH^+^ neurons in LC (*n* = 4, 3 sections from each rat). **f** CPA score recording on test days in EYFP and ChR2 conditioning rats (*n* = 6/group; ***p < 0.0001, two-way ANOVA with Bonferroni test). **g** The effects of propranolol infusion on CPA score during opto-activation of LC neurons (blue light pulse 10 ms, 20 Hz frequency, 3 min ON and 3 min OFF; *n* = 6/group; ***p < 0.0001, two-way ANOVA with Bonferroni test). **h** Effect of optogenetic stimulation of ChR2 on U-69593-induced CPA (*n* = 6/group; *p* = 0.6748, two-way ANOVA). **i** Representative images of c-Fos expression level in LC and ACC regions. Scale bar: 50 µm. **j** Quantitative analysis of c-Fos expression in EYFP, ChR2-saline, and ChR2-propranolol rats (*n* = 3 rats/group, three sections from each animal; ***p < 0.0001, **p < 0.001, ^ns^*p* = 0.8677, one-way ANOVA with Tukey’s test). Results are expressed as mean ± SEM. ns = non-significant, *p* > 0.05. Propran = propranolol.

To specifically activate LC neurons projecting to ACC during conditioning, rats were injected with AAV2-retro-Cre into ACC and AAV2/9-Dio-ChR2-EYFP into LC (Fig. 4a). Together, these vectors produced an expression of ChR2-EYFP only in LC neurons projecting to ACC with efficiency >26.5% and specificity >94% (Fig. 4c–e, Supplementary Fig. 1b, and Supplementary Data 1). On test days, optogenetic activation of noradrenergic neurons during training and before testing days significantly increased the CPA score (Fig. 4b, f, Supplementary Fig. 1f, Supplementary Table 4, and Supplementary Data 1). Further, we report that optical stimulation of noradrenergic neurons in the absence of CRD does not affect the CPA score (Supplementary Fig. 3a, c and Supplementary Data 1).

Next, we performed the activation of noradrenergic neurons with the infusion of propranolol into ACC. We identified injection of propranolol into ACC before conditioning substantially reduced the CPA score (Fig. 4g, Supplementary Table 4, and Supplementary Data 1). In addition, optogenetic stimulation of LC neurons had no effects on U69593-induced CPA (Fig. 4h, Supplementary Table 4, and Supplementary Data 1). Whereas the photo-stimulation of noradrenergic neurons did not change the visceromotor responses to CRD (Supplementary Fig. 2a, b and Supplementary Data 1).

To gain insight into changes in the LC neurons projecting to ACC and within ACC itself, we collected the brains 90 min following activation of LC neurons and stained c-Fos. Notably, light activation of noradrenergic neurons along with propranolol infusion into ACC before conditioning significantly decreased c-Fos expression in the ACC compared to EYFP and ChR2-saline rats (*p* < 0.0001, F_(2, 24)_ = 31.37 (LC); *p* < 0.0001, F_(2, 24)_ = 32.78 (ACC); Fig. 4i, j and Supplementary Data 1). In a separate group of animals, we found that optical stimulation of ACC projecting LC neurons before testing day 1 significantly upregulated the c-Fos expression in the LC and ACC region respectively (*p* = 0.0011, F_(2, 9)_ = 16.09 (LC); *p* = 0.0004, F(2, 9) = 21.73 (ACC); Supplementary Fig. 4a–c and Supplementary Data 1).

Taken together, we demonstrated real-time opto-activation of ACC projecting LC neurons facilitates aversive memory with the recruitment of the ACC neurons during learning, which is disrupted by injection of propranolol.

### Optogenetic activation of β2ARs receptors in the ACC astrocytes facilitates aversive memory and induces the learning-dependent plasticity changes

To demonstrate whether ACC astrocytes play an important roles in aversive memory, we first bilaterally injected AAV-GFAP-ChR2-EYFP and implanted fibres in ACC. The ChR2-EYFP is expressed explicitly in ACC astrocytes with higher efficiency (>95%) and specificity (>97%; Supplementary Fig. 1c, d and Supplementary Data 1). We found that optogenetic activation of ACC astrocytes during conditioning significantly promoted the CPA score (Supplementary Fig. 1e, Supplementary Table 5, and Supplementary Data 1). Additionally, we report that optical stimulation of ACC astrocytes in the absence of CRD does not change the CPA score (Supplementary Fig. 3a, d and Supplementary Data 1).

Next, we validated that β2ARs are also expressed in the rat ACC astrocytes (Supplementary Fig. 5a). Moreover, no significant difference in the expression level of ACC astrocytic β2ARs was detected between control and CRD-conditioned rats (Supplementary Fig. 5a). However, recent studies involving large-scale sequencing and splicing databases showed that β2ARs are expressed in microglia whereas β1ARs are also expressed in astrocytes^27,41^. Our results corroborated the findings of these researchers (Supplementary Fig. 5a, b).

**Fig. 5:**
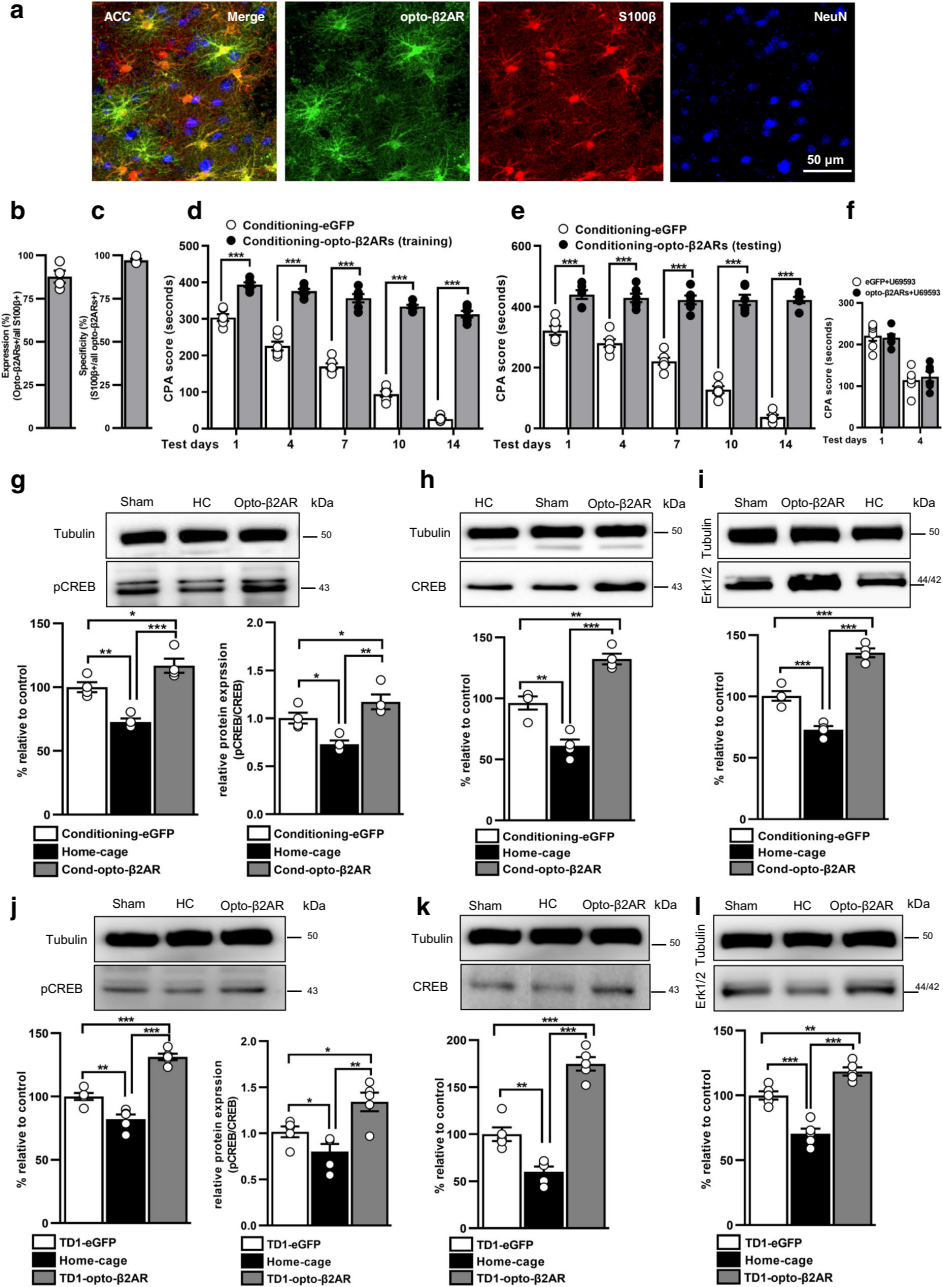
Optogenetic activation of ACC astrocytic β2ARs promotes aversive memory. **a** Representative images of opto-β2AR (green) in the ACC astrocytes. Scale bar: 50 µm. % expression (**b**) and specificity (**c**) of opto-β2AR-eGFP in ACC astrocytes (*n* = 4, 3 sections from each rat). CPA score in eGFP and opto-β2AR activation rats when optogenetic activation was performed during training (**d**) and before testing days (**e**) respectively (blue light pulse 50 ms, 10 Hz frequency, 3 min ON and 3 min OFF; *n* = 6/group; ***p < 0.0001, two-way ANOVA with Bonferroni test). **f** Effect of optogenetic activation of β2AR on U-69593-induced CPA (*n* = 6/group; *p* = 0.5651, two-way ANOVA). Representative images and quantitative western blot analysis for pCREB (**g**), pCREB/CREB (**g**), CREB (**h**), and Erk1/2 (**i**) from ACC extract of eGFP and opto-β2AR-eGFP rats, opto-activated during conditioning and sacrificed 90 min after training and opto-activated before testing day 1 and sacrificed 90 min after testing (**j**–**l**) (*n* = 4–5/group; ***p < 0.0001, **p < 0.001, *p < 0.01, one-way ANOVA with Tukey’s test). Results are presented as protein percentage of control sample mean values (100%). Protein values are normalized to those of tubulin. All results are presented as mean ± SEM. ns=non-significant, *p* > 0.05. TD1 = Test day 1. HC = home cage.

To confirm the effects of astrocytic β2ARs in aversive memory, we took advantage of the chimeric rhodopsin/opto-β2ARs. Stereotactic injection of AAV2/5-gfaABC1D-opto-β2AR-eGFP into ACC region resulted in >87% S100β+ cells expressed opto-β2AR+ with a specificity of >97% (Fig. 5a–c and Supplementary Data 1). On test days, optogenetic activation of astrocytic β2ARs during training or before testing days significantly promoted the CPA score (Fig. 5d, e, Supplementary Table 5, and Supplementary Data 1). In addition, we showed opto-activation of β2ARs in ACC astrocytes had no effects on U69593-induced CPA (Fig. 5f, Supplementary Table 5, and Supplementary Data 1). Further, optical stimulation of ACC astrocytic β2ARs in the absence of CRD does not change the CPA score (Supplementary Fig. 3a, e and Supplementary Data 1). In separate group of rats, we also showed that photoactivation of astrocytic β2ARs did not change the VMR to graded pressure of CRD (Supplementary Fig. 2c, d and Supplementary Data 1).

Next, to test whether photo-stimulation of astrocytic β2ARs can induce a learning-dependent increase in plasticity changes, we collected the brains and performed a western blot. Light activation of ACC astrocytic β2ARs during training or before testing day 1 significantly increased the expression level of pCREB, pCREB/CREB, CREB, and Erk1/2 compared to eGFP and home cage rats (*p* < 0.0001; Fig. 5g–l and Supplementary Data 1).

Consistent with previously published reports^42^, the present data demonstrate that optogenetic stimulation of astrocytic β2ARs induce memory-related increase in cellular changes known to underlie synaptic plasticity.

### Cell-specific knockdown of β2ARs in ACC astrocytes, not neuronal β2ARs, suppresses aversion learning and memory

**Fig. 6:**
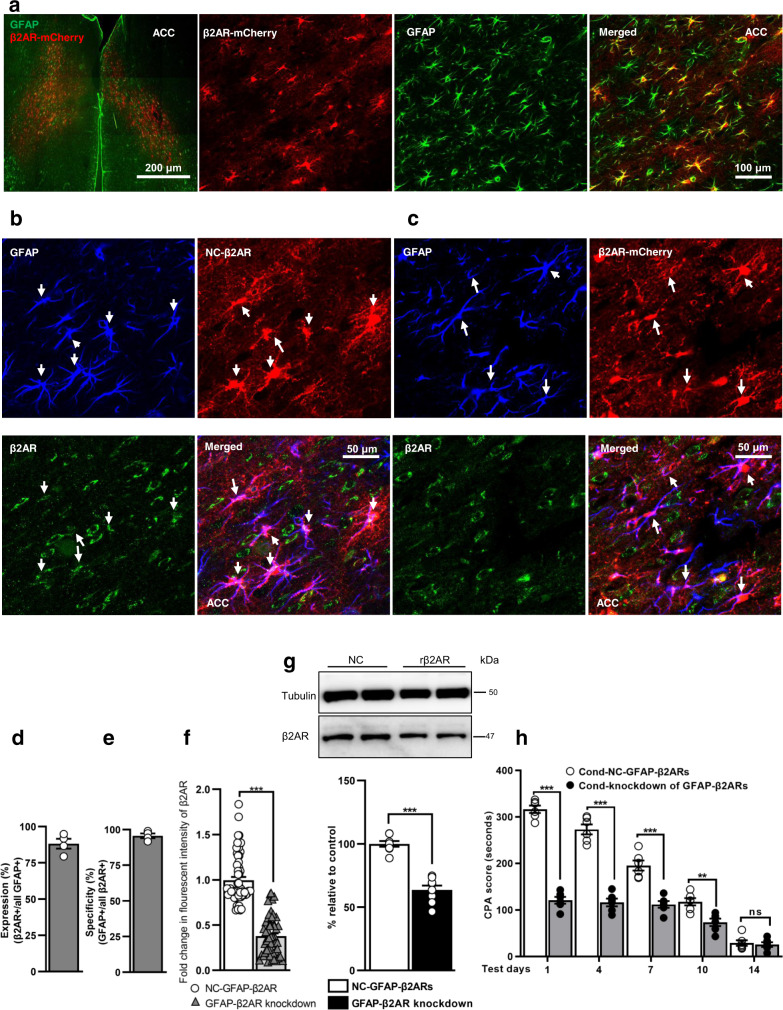
Cell-specific knockdown of β2ARs in ACC astrocytes suppresses aversion learning memory. **a** Representative images of AAV2/5-gfaABC1D-mCherry-miRNAi(rβ2AR) in the ACC astrocytes. Scale bar: 200 µm, 100 µm. Representative images of negative control (**b**) and β2AR-mCherry virus (**c**; red) expression in ACC astrocytic β2AR (green). Scale bar: 50 µm. % expression (**d**) of virus transfection and specificity (**e**) in ACC astrocytes (*n* = 4/group, 3 sections from each rat). **f** Quantification of fold change in β2AR fluorescent intensity (*n* = 4/group, 3 sections from each rat; *** *p* < 0.0001, Mann–Whitney test). **g** Representative blot images and densitometric analysis for β2AR expression following a knockdown (*n* = 5–8/group; ***p < 0.0001, *t*_14_ = 8.71, unpaired t-test). Results are presented as protein percentage of control sample mean values (100%). Protein values are normalized to those of tubulin. **h** Knockdown effect of β2AR on CPA score (*n* = 6/group; ***p < 0.0001, **p < 0.001 ^ns^*p* = 0.9994, two-way ANOVA with Bonferroni test). All results are expressed as mean ± SEM. ns=non-significant, *p* > 0.05.

To identify the selective functional role of astrocytic β2ARs in aversive memory, we sought to genetically knockdown the astrocytic β2ARs within ACC region using microRNA-based RNA interference (miRNAi) technique delivered by AAV. The immunostaining revealed that >89 % GFAP+ cells in ACC area expressed rβ2AR-mCherry with >96% specificity (Fig. 6a, d, e and Supplementary Data 1). Moreover, co-staining with microglia activation marker Iba1 showed no overlap with β2AR mCherry^+^ cells (Supplementary Fig. 6a). When co-stained with the neuronal nuclear marker NeuN, it offered approximately 1.84% off-target expression in the ACC neurons (Supplementary Fig. 6b, c and Supplementary Data 1).

Using immunostaining, we found that rβ2AR-miRNAi injection resulted in a significant downregulation of β2AR expression in ACC astrocytes (Fig. 6b, c, f and Supplementary Data 1). The western blot data further confirmed that the knockdown efficiency of ACC astrocytic β2ARs was more pronounced in miRNAi(rβ2AR) compared to control (Fig. 6g and Supplementary Data 1). Additionally, immunostaining and western blot data revealed that knockdown of astrocytic β2ARs did not affect the expression of β1ARs in ACC astrocytes (Supplementary Fig. 5c–f and Supplementary Data 1). On test days, knockdown of β2ARs significantly reduced the CPA score compared to control (Fig. 6h, Supplementary Table 6, and Supplementary Data 1). The knockdown of astrocytic β2ARs did not change the VMR to colorectal distension (Supplementary Fig. 2e, f and Supplementary Data 1).

Next, to demonstrate the role of ACC neuronal β2ARs in aversive memory, we used the same approach to knockdown the ACC neuronal β2ARs. The immunostaining showed >88% ACC NeuN+ cells expressed β2AR-mCherry with >98% specificity (Supplementary Fig. 7a, d, e and Supplementary Data 1). The histochemical staining shown that injection of miRNAi into ACC region produced significant reduction in the expression of neuronal β2ARs (Supplementary Fig. 7b, c, f and Supplementary Data 1). The western blot data further confirmed that knockdown effect was more robust in miRNAi(rβ2AR) compared to the negative control rats (Supplementary Fig. 7g and Supplementary Data 1). Notably, on test days, knockdown of ACC neuronal β2ARs has no significant effect on the CPA score (Supplementary Fig. 7h, Supplementary Table 6, and Supplementary Data 1). Intriguingly, taken together, the data demonstrate that ACC astrocytic β2ARs, not the neuronal β2ARs, are required for aversive memory formation.

Next, to test whether astrocytic β2ARs affect spatial memory and anxiety, we conducted Morris water maze task and open field test. Interestingly, knockdown of astrocytic β2ARs did not show any change either in spatial memory or anxiety-like behaviour (Supplementary Fig. 8 and Supplementary Data 1).

### Gi pathway activation disrupts the enhanced aversive memory induced by optogenetic activation of LC neurons and suppresses learning-dependent plasticity changes

First, we tested Gi activation in ACC astrocytes to modulate the acquisition and expression of aversive memory. Stereotactic injection showed that >87% of GFAP+ cells in the ACC region expressed hM4Di-mCherry with a specificity of >94% (Supplementary Fig. 9a–c and Supplementary Data 1). When co-stained with neuronal nuclear marker, <5.5% of hM4Di-mCherry^+^ cells overlapped with ACC neurons (Supplementary Figure 6d, e and Supplementary Data 1). On test days, activation of the Gi pathway in ACC astrocytes before training or before testing days substantially blocked the CPA memory (Supplementary Fig. 9d, e, Supplementary Table 7 and Supplementary Data 1). In a separate group of rats, we also showed that the CNO (1 mg/kg b.w) treatment before conditioning itself does not affect the CPA score and c-Fos expression in the ACC region (Supplementary Fig. 10a–d and Supplementary Data 1).

Next, we measured learning-dependent plasticity changes after activation of astrocytic Gi pathway. Strikingly, the western blot showed a significant decrease in the expression level of pCREB (*p* < 0.0001, F_(3, 16)_ = 19.68), pCREB/CREB (*p* < 0.0001, F_(3, 16)_ = 21.93), CREB (*p* < 0.0001, F_(3, 16)_ = 21.18), and Erk1/2 (*p* < 0.0001, F_(3, 16)_ = 43.44), in the CNO injected rats (before training) compared with saline, home cage-saline, and home cage-CNO rats (Supplementary Fig. 9f–h and Supplementary Data 1). Similarly, reduced expression of pCREB (*p* < 0.0001, F_(3, 13)_ = 62.27), pCREB/CREB (*p* < 0.0001, F_(3, 13)_ = 9.36), CREB (*p* < 0.0001, F_(3, 16)_ = 17.93), and Erk1/2 (*p* < 0.0001, F_(3, 16)_ = 25.79) was observed in the CNO injected rats on testing day 1 compared to saline, home cage-saline, and home cage-CNO rats (Supplementary Fig. 9i, j, k and Supplementary Data 1). However, Gi pathway activation did not affect the CRD-induced VMR (Supplementary Fig. 2g, h and Supplementary Data 1). We report Gi pathway activation impairs the aversive memory formation due to disruption in memory-related plasticity changes.

**Fig. 7:**
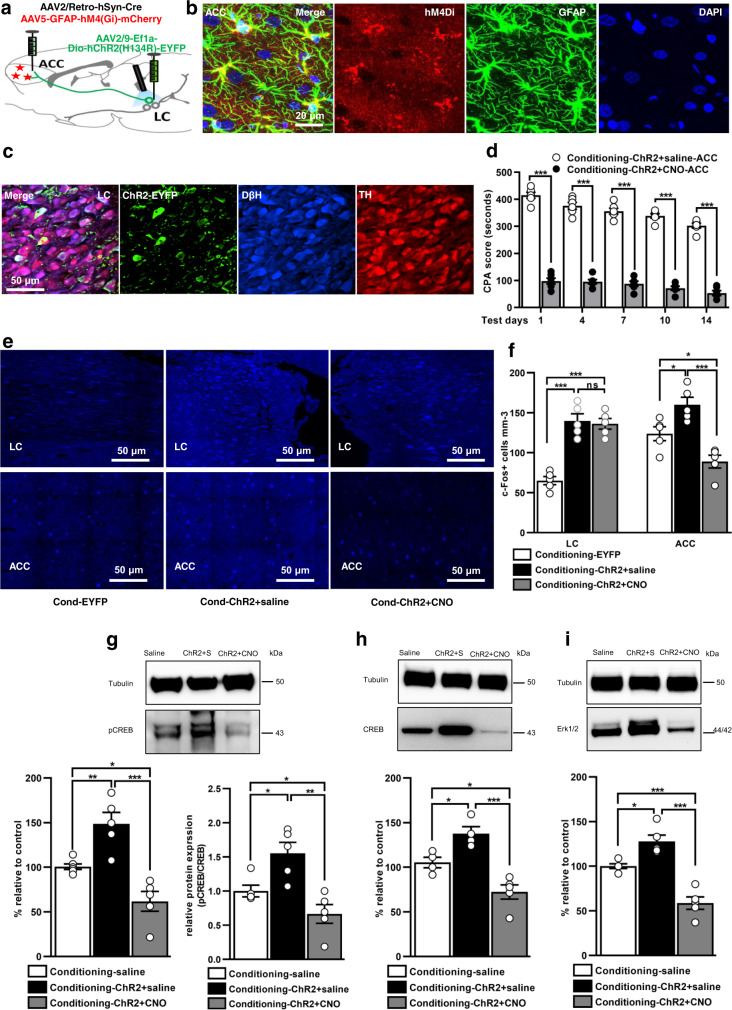
Astrocytic Gi pathway activation disrupts enhanced aversive behaviour induced by opto-stimulation of LC neurons and suppresses the expression of plasticity changes. **a** Schematic protocol for three viral constructs. **b** Representative images of hM4Di viral expression in ACC astrocytes. Scale bar: 20 µm. **c** Expression of Dio-ChR2 in Th^+^ and DβH^+^ neurons of LC region. Scale bar: 50 µm. **d** CPA score in ChR2-saline and ChR2-CNO injected rats on test days (blue light pulse 10 ms, 20 Hz frequency, 3 min ON and 3 min OFF; *n* = 6/group; ***p < 0.0001, two-way ANOVA with Bonferroni test). **e** Representative images of c-Fos expression in LC and ACC regions. Scale bar: 50 µm. **f** Quantification of c-Fos in EYFP, ChR2-saline, and ChR2-CNO rats (*n* = 5 rats/group, three sections from each animal; ***p < 0.0001, *p < 0.01, ^ns^*p* = 0.9313, one-way ANOVA with Tukey’s test). Representative blot images and western blot analysis of pCREB (**g**), pCREB/CREB (**g**), CREB (**h**), and Erk1/2 (**i**) in saline, ChR2-saline and ChR2-CNO rats (*n* = 4–6/group; ***p < 0.0001, **p < 0.001, *p < 0.01, one-way ANOVA with Tukey’s test). Results are presented as protein percentage of control sample mean values (100%). Protein values are normalized to those of tubulin. All results are presented as mean ± SEM. ns=non-significant, *p* > 0.05.

Next, we hypothesized Gi activation in astrocytes could prevent the recruitment of ACC during optogenetic activation of LC neurons. To test the hypothesis, rats were injected with AAV2-retro-Cre and AAV-GFAP-hM4D(Gi)-mCherry into ACC region and a Cre-dependent Dio-ChR2-EYFP into LC, causing the expression of hM4D(Gi)-mCherry in ACC astrocytes and ChR2-EYFP in ACC projecting LC neurons (Fig. 7a–c). On test days, activation of astrocytic Gi pathway before training substantially disrupted the increased aversion memory induced by opto-activation of ACC-projecting LC neurons (Fig. 7d, Supplementary Table 7, and Supplementary Data 1). Surprisingly, photoactivation of LC neurons and simultaneous Gi pathway activation in ACC astrocytes significantly reduced the c-Fos expression in the ACC compared to EYFP and ChR2-saline rats (Fig. 7e, f and Supplementary Data 1; F_(2, 12)_ = 35.67 (LC); F_(2, 12)_ = 16.80 (ACC).

Lastly, western blot analysis revealed a significant reduction in the expression of pCREB (*p* < 0.001, F_(2, 12)_ = 18.80), pCREB/CREB (*p* < 0.0001, F_(2, 12)_ = 11.61), CREB (*p* < 0.01, F_(2, 10)_ = 21.94), and Erk1/2 (*p* < 0.0001, F_(2, 12)_ = 34.92) in the ChR2-CNO group compared to saline and ChR2-saline (Fig. 7g–i and Supplementary Data 1). These observations suggest that ACC astrocytic Gi manipulation produced a promising effect on ACC neuronal activity but has no influence on LC activity itself. The treatment effectively prevented successful engagement of ACC and suppressed learning-dependent molecular changes that underlie synaptic plasticity.

## Discussion

The biological phenomenon of pain is highlighted by its conscious experience and subjectivity. In recent years, numerous studies have established that threshold and magnitude of pain can be readily modulated by interactions between memory, attentional, and affective brain circuitry^12–14^. Pain is comprised of sensory and affective elements; evidence indicates that nociceptors can function without pain perception^1,43^. Clinical data has indicated that the pain-induced emotional or motivational effects may be endured much longer than the pain itself. Here we ask how acute visceral nociceptive stimulation results in a prolonged negative affective state in rodents and explore the neural processing and circuitry mechanisms governing pain-evoked aversive learning and memory.

The studies conducted on human and animal models have suggested that the anterior cingulate cortex (ACC) is a key area that receives multiple inputs and its related structures are important in relaying and processing pain perception^11,44,45^. Using a visceral hypersensitivity rat model, our previously published data characterized the impaired synchronization in ACC neural circuitry and cognitive deficits in the chronic visceral pain state^2–6,46^.

Previous reports have identified that ACC is essential for the aversive nature of nociceptive stimulation^7,8,15,16^. Avoidance behaviour is exhibited in animals to avoid potential negative outcomes. The majority of rodent studies on pain-conditioned passive avoidance have used noxious colorectal distension (CRD 60 mmHg)^12^ or high-intensity noxious laser stimulation^13^. During retrieval of pain-conditioned passive avoidance, acute pain processing was recruited. In this study, combining CRD^5^ with conditioned place avoidance (CPA) paradigm, we demonstrated that when CRD (≤35 mmHg, a sub-threshold for pain perception) was paired with a distinct environmental context, rats spent significantly less time in this compartment during the post-conditioning test. These observations suggest that, upon experiencing visceral nociceptive stimulation, rats develop considerable aversion to the location associated with sub-threshold for pain^15,16^. The nociceptive stimulus given during CRD-CPA tests result in a negative affective state in the animal and elicits acute nociceptive behaviours such as lifting and licking. Here, we confirm these findings and show that the aversive behaviour remained for two more weeks. We have reported that ACC lesions or microinjection of an excitatory amino acid antagonist into the ACC during conditioning blocked learning triggered by a noxious stimulus and reduced CPA scores, suggesting that the ACC is essential for the aversive nature of visceral nociceptor stimulation^15,16^. How do the affective dimensions of pain persist much longer than the pain sensation itself?

The LC contains extensive projections throughout the neuraxis and plays a crucial role in cognitive processes such as learning and memory^47^, and arousal^48,49^. The functioning of the prefrontal cortex is heavily sensitive to LC-derived norepinephrine^21,50^, and can be significantly altered by low levels of norepinehrine^51^.

Although the LC is conventionally thought to be a pain suppressor^52^, a review contends that the LC transforms into a chronic neuropathic pain generator after traumatic nerve injury^53^. A study by Hirschberg et al.^54^ reported that hind-limb sensitization was modulated by noradrenergic neuronal populations in the spinal cord (LC^:SC^), while the pain-aversion was modulated by a distinct area in prefrontal cortex (LC^:PFC^)^54^. Here, we observed that selective depletion of LC noradrenergic neurons by immunotoxic agent anti- DβH saporin^55,56^ markedly suppressed the pain-evoked aversive learning and memory.

We examine the effects of LC lesion on CPA induced by an aversive, but non-nociceptive-activating stimulus. Mu-opioid receptor agonists, which function as rewarding stimuli are known to be non-noxious, whereas kappa-opioid receptor agonist U69,593 has been established as an aversive stimuli^8,57,58^. These motivational effects have been attributed to interactions of exogenous opioids with endogenous reward pathways in the brain^59^ when injected systemically and paired with a distinct compartment in the apparatus. The conditioning procedure was similar to that used the CRD-induced CPA. Consistent with our previously published data, we showed that the depletion of LC noradrenergic neurons had no effect on aversive responses to the k-opioid receptor agonist U69,593-induced CPA^15,16^. The depletion of LC noradrenergic neurons was observed to have no effect on pain sensation (CRD-induced VMR), suggesting that the LC-norepinephrine system does not play a role in modulating sensory component of visceral pain perception in the normal physiological state.

In humans and rodents, pharmacological studies utilizing βARs agonists and antagonists have suggested that βARs play an important role in encoding, modulation and retrieval of memory^60^. β1ARs one of the subtypes of β-adrenergic receptors, are expressed predominantly at the pre- and post-synaptic terminals of neurons^20^. Studies on animal models lacking β1ARs, or treated with selective β1AR agonists or antagonists have demonstrated the role of synaptic plasticity, as well as memory formation and retrieval^61,62^. β2ARs, which are primarily expressed in glia^19,37^, are vital to the modulation of amygdala-dependent memory and the functioning of hippocampus and prefrontal cortex^63^. Studies have showed that inhibitors of βARs, such as propranolol disrupted memory consolidation and strengthening^20^. In addition, genetic deletion of β2ARs leads to the impaired modulation of amygdala-dependent memory by stress or corticosterone and disrupted hippocampal plasticity^64^. β2ARs antagonists ICI118,551 binds to the β_2_ subtype with at least 100 times greater affinity than β1 or β3^65^. A previous study has showed that bilateral injection of ICI 118,551 into the hippocampus before training the impaired long-term memory^60^. We report that bilateral infusion of βARs antagonist propranolol and selective β2ARs antagonist ICI118,551 into ACC disrupted the pain-related aversive learning and memory via reduction in the expression level of synaptic plasticity and memory-dependent molecular changes pCREB, CREB expression, as well as Erk1/2^66,67^. These results support the hypothesis that visceral noxious stimuli activates LC neurons to release neuromodulators in the cortex, which then rapidly alters LC-ACC network communication. This brain network processing is necessary for encoding, consolidation and retrieval of a long-term post-visceral pain aversive memory component compared to fast-acting, acute pain sensation.

The use of chemogenetic and optogenetic tools in neuroscience research allows continuous and reversible manipulation of astrocyte populations in combination with behavioural measurements^48,68^. Given that minute changes of norepinehrine activity in the PFC can result in significant effects on cognitive functions, we applied a retrograde tracing technique combined with optogenetic stimulation to specifically activate the LC neurons projecting to ACC. The ACC area was defined as the cingulate cortex, area 2 (Cg2) and prelimbic cortex together with the overlying cingulate cortex, area 1 (Cg1)^2^. Enhanced aversive behaviour was observed to be blocked by propranolol. These data indicate that norepinephrine derived from the bottom-up LC to ACC neuronal pathway is responsible for inducing aversive behaviour. Next, optogenetic inhibition of LC neurons during the training days or before memory test abolishes the CPA responses, suggesting inhibition of the aversive learning consolidation and disrupted memory recall. On the other hand, optogenetic silencing of LC neurons had no effect on pain sensation (CRD-induced VMR). It appears that distinct mechanisms modulate sensory and aversive component of visceral nociceptive stimulation in the normal physiological state. However, the mechanism governing the LC input to prefrontal cortex in regulating complex pain-related aversive processes is still unknown.

Astrocytes are able to differentiate the activity of discrete synapses that originate from different afferents and modify synaptic activity at surrounding synapses by releasing active substances^22^ to modulate various neuronal circuits^30,36,69–71^, and affect behavioural responses^29,38,72,73^. It has been well demonstrated that astrocytes support neuronal functions by regulating the flow of extracellular ions and neurotransmitters, and by providing neuronal energy substrates such as lactate by means of their astrocytic processes. Our recent study showed that optogenetic activation of astrocytes in the ACC triggers lactate release, improves decision making in normal rats and rescues the decision-making impairment in rats with chronic visceral pain^32^. Activation of astrocytes further rescues ACC synaptic LTP and repairs impaired spike phase locking in rats^32^. β2ARs activation promotes glucose uptake may regulate astrocyte glucose metabolism, such as lactate production or transduction^74^. It is reasonable that during CPA training and memory retrieval, the increased brain activity may lead to glutamate release, which enter astrocytes in a Na^+^-dependent manner leading to the activation of Na+/K+-ATPase pump, which promotes the consumption of glucose, glycolysis, and production of L-lactate^75^. Astrocytes are the main sites of glycogen storage in the CNS,  and can rapidly metabolize glycogen to lactate^76^ and transport it to neurons during periods of neuronal activity. Recently, we have demonstrated that astrocyte-lactate signalling in the ACC is required in regulating visceral aversive memory^77^.

βARs are expressed in neurons and in other cell types, such as astrocytes. An intriguing open question is whether LC neurons project to distal areas of cortex where they release norepinephrine triggering astrocyte signals^34^. We hypothesize that the network activities of LC-ACC noradrenergic projection in memory consolidation is mediated by astrocyte signalling. First, we confirmed that stereotactic viral injection of the AAV8-GFAP::ChR2(H134R)-EYFP virus into ACC resulted in specific viral expression in ACC astrocytes. We showed optogenetic astrocytic modulation in the ACC during the conditioning/training significantly facilitated the pain aversive learning, consolidation and memory recall. It is acknowledged here that optogenetic activation of astrocytes via channel rhodopsin 2 (ChR2) does not mimic a specific characteristic of physiological effects of the astrocytes. Further studies are required to clarify the mechanisms of ChR2 activation in astrocytes. Next, we expressed the Gi-coupled receptor hM4Di in ACC astrocytes. We used chemogenetic DREADD receptor technology to manipulate ACC astrocytic activity through Gi pathway activation via hM4Di receptor^30,78^. In the present study <5.5% of hM4Di receptors were expressed in the neurons. This accounts for only <2.5% of the total ACC neurons and this amount of neurons alone would not be able to drive any significant effects. ACC astrocytic Gi activation during learning and consolidation was sufficient to specifically block the nociceptive aversive memory induced by opto-stimulation of projection-specific LC neurons, but did not affect pain sensation measured by noxious colorectal distention. These findings point to the importance of ACC astrocytes in aversive learning and memory processes.

An important issue is whether astrocytes norepinephrine signalling can be expressed and activated in specific receptors during aversive learning. We performed a study by stereotactic viral injection of AAV2/5-gfaABC1D-opto-b2AR-eGFP into ACC, which resulted in specific viral expression in ACC astrocytes as shown by GFAP cells expressed opto-β2AR^+^ cells. We show that optogenetic manipulation of β2ARs receptors in ACC astrocytes promotes conditioned place aversion memory. Astrocytes exhibit task-specific effects in vivo^79^. Accumulating but still limited evidences indicated that astrocytes influence neuronal activity through mechanisms including the homoeostatic control of extracellular levels of ions and neurotransmitters^36,69^. In the present study, we did not identify the gliotransmitter engaged in the activation of astrocytic β2 receptors. Further studies are needed to investigate the molecular mechanisms involved in astrocyte-neuron interactions at the cellular level.

In this study, conclusive evidence is provided usin*g* virus-mediated, cell-specific knockdown approach^37^. The rats were injected with AAV encoding β2AR miRNAi with the astrocytic GFAP promoter into ACC to knockdown β2AR. Using the microRNA silencing technique with GFAP promoter and hsyn promoter, we found that knockdown astrocytic but not neuronal β 2ARs in ACC can impair CPA memory, indicating that β2ARs expressed by the ACC astrocytes rather than neurons may be the critical effectors of the adrenergic-mediated effect on aversive memory formation. Our results are consistent with previous findings concerning the function of astrocytic β2ARs^60^. Gao et al.^60^ found that shRNA-mediated knockdown of β2AR in astrocytes but not in neurons in HPC impaired inhibitory avoidance long-term memory^60^. However, as ACC consists of several types of neurons, including excitatory neurons, somatostatin, parvalbumin, calretinin, and calbindin interneurons, whether β2ARs expressed by the specific neuronal subtypes have an important role in aversive memory formation has not been addressed in this study and requires further investigation.

In another finding, we found that ACC astrocytic Gi activation during memory acquisition impairs aversive memory induced by direct optogenetic activation of specific LC neurons projecting to ACC, which consequently prevents the expression of plasticity genes in ACC. These data indicate that ACC astrocytes can produce specific effects on surrounding neurons by modulating neuronal activity, and astrocytic signalling in the ACC network is necessary for promoting synaptic plasticity and network communication.

To summarize, we demonstrate that network activities of LC-ACC noradrenergic projections modulate the encoding of sub-thresholds pain-related negative affect that support associative learning and avoidance. Over time, ACC astrocytic β2AR activation may play a key role in manifesting pain-related behaviour based on previous aversive learning.

In conclusion, our findings establish a previously unknown, LC-ACC neuron-astrocyte circuit mechanism specific to visceral aversive avoidance behaviour, revealing potential targets for therapeutic interventions in chronic pain-induced pathological disorders of behaviour.

## Methods

### Experimental model and subject details

#### Animals and ethical consideration

All the experimental work was carried out on adult male Sprague Dawley (SD) rats weighing about 250–300 grams. They were kept in cages with 24 h access to food chow, and water. The animals were maintained in a holding room with a constant room temperature of 25 °C and a 12:12 h light and dark cycle. Animal studies were performed in accordance with the guidelines laid down by the Committee on the Use and Care of Animals, Department of Health, Govt. of Hong Kong SAR [Animals (Control of Experiments) Ordinance (Cap. 340), License to Conduct Experiments Ref: (19–155) in DH/HT&A/8/2/5 Pt. 1, (19–157) in DH/HT&A/8/2/5 Pt. 1 and (20–16) in DH/HT & A/8/2/5 Pt. 1]. Approvals for “Ethical Review of Research Experiments involving Animal Subjects” were granted by Animal Research Ethics Sub-Committee, City University of Hong Kong (Ref: A-0557).

### Method details

#### Depletion of LC noradrenergic neurons

The male SD rats, after deep anaesthesia with intra-peritoneal injection of sodium pentobarbital (40 mg/kg BW), underwent local bilateral injections of the anti-DβH-saporin immunotoxin (Millipore catalogue No. MAB394) into the locus coeruleus (LC). Briefly, 0.25 μg of anti-DβH saporin (1 μg/μl) dissolved in sterile phosphate-buffered saline^55,56^ was injected with a ten μl microsyringe with a 33-gauge metal needle (Hamilton, NV, USA), controlled by a microsyringe pump (World Precision Instruments, FL, USA), into each hemisphere of LC. The following coordinates was used: AP = −9.8 mm (caudal to bregma); ML = ±1.2 mm (lateral to the midline); DV = −7.0 mm (ventral to the dura)^80^. Each infusion was carried out over 3 min at a flow rate of 0.17 μl/min, kept the injector in place for another 3 min to prevent backflow of the fluid, and slowly withdrawn to minimize the tissue damage. Vehicle injections with the exact coordinates, volume, and speed were used for control animals. This volume of drug generated more than 90% ablation of LC noradrenergic neurons and complete loss of DβH immunoreactive cell bodies throughout LC. The immunotoxin depletion of LC noradrenergic neurons did not affect other non-adrenergic neurons or their efferent projections to the respective targets^55^.

#### Cannula implantation and drug delivery

The rats were anesthetized with general anaesthetic sodium pentobarbital (40 mg/kg i.p.). Stainless steel guide cannulae (26-gauge) were bilaterally positioned in the ACC region based on the following coordinates AP = +2.2–3.8 mm, ML = ±0.5–1.0 mm, DV = 1.5–3.5 mm from the skull surface. The guide cannulae were fixed with the help of screws and dental cement (Megadental, Germany) to the skull. Dummy cannulae 0.5 mm longer than the guide cannulae were placed into the guide cannulae to prevent blockage and reduce the risk of infection. The rats were provided a minimum recovery period of one week before the experimental procedures were performed.

Following drugs were used and purchased from Sigma unless otherwise stated. Propranolol hydrochloride (5 µg/µl; Sigma-Aldrich, Catalogue No. P0884)^60^ dissolved in PBS containing 10% DMSO. ICI 118,551 hydrochloride (5 µg/µl per side; Sigma-Aldrich, Catalogue No. 505275)^60^ was mixed in 0.9% saline or aCSF. All the drugs were injected 15 min before training where appropriate, and infusions were performed at 1 µl of volume per ACC side. The infusion needles extended 0.5 mm beyond the guide cannula. The rats received bilateral ACC injections at indicated time points before or after the training, at a rate of 0.333 ml/min^32^ with an infusion pump (World Precision Instruments, USA). The micro-injection needle was kept in place for an additional 3 min following the injection to allow for the complete dispersion of the solution. U-69593 (Sigma-Aldrich, Catalogue No. U103) is a non-noxious selective kappa-opioid receptor agonist known for its aversive response (0.16 mg/kg of body weight; s/c)^15,16,58^. Clozapine N-oxide hydrochloride (CNO; 1 mg/kg of BW; Sigma-Aldrich, Catalogue No. SML2304) was used to activate the DREADDs hM4D(Gi) receptors.

#### Virus injections and fibre implantation

The rats were anesthetized with general anaesthetic sodium pentobarbital (40 mg/kg i.p.), and their head were fixed into stereotactic apparatus (Kopf Instruments, USA). The skull was exposed, and AAV2-retro-hSyn-Cre (Vigene Biosciences, China, 9.0 × 10^13^ virus molecules/ml, diluted 1:5 in sterile PBS, 0.400 µl per site) was injected into ACC. In the same group of rats, either photoinhibition Cre-dependent viral construct AAV2/9-Ef1α::Dio-eNPHR3.0-EYFP (Taitool Bioscience, Shanghai, China, 1.0 × 10^13^ virus molecules/ml, diluted 1:10 in sterile PBS, 0.250 µl per site) or photostimulation Cre-dependent viral construct AAV2/9-Ef1α::Dio-hChR2(H134R)-EYFP (Taitool Bioscience, Shanghai, China, 1.27 × 10^13^ virus molecules/ml, diluted 1:10 in sterile PBS, 0.250 µl per site), was injected into each hemisphere of LC with 10 µl Hamilton Syringe. The flow rate of microinjection (0.1 μl/min) was controlled by a microinjection pump (World Precision Instruments, USA). The following stereotaxic coordinates, 9.8 mm caudal, 1.2 mm lateral from bregma, and 7.0 mm ventral from the surface of the skull was used^80^. The needle was kept in the target site for additional 5 min to allow the proper diffusion of the virus and slowly withdrawn. The AAV-retro induced the expression of Cre recombinase into ACC and the Cre-dependent viral construct induced the expression of EFYP into the LC region. After viral injection, rats received bilateral surgical implantation of the chronic fibre-optic cannula at 0.3 mm above the injection site (core diameter 200 μm and numerical aperture 0.39 NA; Thorlabs, USA). The control groups were infected with the same volume of control virus without photoinhibition or photo-stimulation construct eNpHR3.0 and ChR2, respectively. They were then embedded with a fibre-optic cannula delivering blue or yellow light into LC. The rats were kept undisturbed for four weeks to recover and allow gene expression. The main advantage of eNpHR and ChR2 over the traditional pharmacological or genetic loss of cellular functions is its capacity to inhibit neural activity at specific time windows with minor distress and damage to the animal^48^.

For optogenetic activation of ACC astrocytes, 1 μl of AAV8-GFAP-ChR2(H134R)-EYFP (5.5 × 10^12^ virus molecules/ml; packaged by UNC Vector Core, University of North Carolina, USA) was injected into each side of the ACC. The flow rate of 0.1µl/min was controlled by a microinjection pump (World Precision Instruments, USA). The needle was kept in the target site for additional 5 min to allow the proper diffusion of the virus and slowly withdrawn. The following stereotaxic coordinates AP = 2.8 mm, 0.8 mm lateral from bregma, and 2.8 mm ventral from the surface of the skull were used^32^. The control group was transfected with the control virus without ChR2(H134R) but still implanted with the fibre optic cannula delivering blue light into ACC. After viral injection, rats received bilateral surgical implantation of the chronic fibre-optic cannula at 0.3 mm above the injection site (core diameter 200 μm and numerical aperture 0.39 NA; Thorlabs, USA). The rats were kept undisturbed for three weeks to recover and allow gene expression. The optogenetic manipulation of astrocytes elevates the Ca2+ levels and activates the astrocytic Ca^2+^-calmodulin signalling and release of ATP. These events are associated with the excitation of astrocytes^31^.

For optogenetic activation of β2ARs, AAV2/5-gfaABC1D-opto-b2AR-eGFP (Addgene plasmid No. 20948; packaged by Taitool Bioscience, China; 1.28 × 10^13^ virus molecules/ml, diluted 1:10 in sterile PBS, 0.8 µl per site), was injected into ACC at a flow rate of 0.1 μl/min using 10 μl Hamilton syringe with 33 G injection cannula. The needle was kept in the target site for additional 5 min to allow the proper diffusion of the virus and slowly withdrawn. The following stereotaxic coordinates AP = 2.8 mm, 0.8 mm lateral from bregma, and 2.8 mm ventral from the surface of the skull were used^32^. The control groups were transfected with the same volume of control virus AAV2/5-gfaABC1D-eGFP-WPRE-pA (1.1 × 10^13^ virus molecules/ml, diluted 1:10 in sterile PBS). The rats received bilateral surgical implantation of chronic fibre-optic cannula after viral injection at 0.3 mm above the injection site (core diameter 200 μm and numerical aperture 0.39 NA; Thorlabs, USA). The rats were kept undisturbed for three weeks to recover and allow gene expression. The optogenetic activation of astrocytic β2AR receptors recruits the stimulatory G protein-coupled receptors pathway, activating the adenylate cyclase enzyme and finally increasing the cAMP level^31,42^. This activation is comparable to that achieved by the pharmacological activation of wild-type (endogenous) β2ARs with β2AR agonists^31,42^.

#### Gi manipulation and light activation of LC neurons

A double viral injection strategy was used to demonstrate that ACC astrocytes Gi manipulation can block the activity of LC neurons projecting to ACC and ACC activity in general. A mixture of AAV2-retro-hSyn-Cre (Vigene Biosciences, China, 9.0 × 10^13^ virus molecules/ml, diluted 1:5 in sterile PBS, 0.300 µl per site) and AAV5-GFAP-hM4D(Gi)-mCherry (Addgene, 50479-AAV5; 1.0 × 10^13^ GC/ml, diluted 1:10 in sterile PBS, 0.7 µl per site) to allow astrocytic manipulation was injected into each side of ACC (AP = 2.8 mm, ML = 0.8 mm and DV = 2.8). The Cre-dependent virus AAV2/9-Ef1α-Dio-hChR2(H134R)-eYFP (Taitool Bioscience, Shanghai, China, 1.27 × 10^13^ virus molecules/ml, diluted 1:10 in sterile PBS, 0.250 µl per site), was injected into LC that allowed the expression of EYFP into LC neurons projecting to ACC. The rats received bilateral surgical implantation of the chronic fibre-optic cannula at 0.3 mm above the injection site in LC. To activate the Gi pathway of ACC astrocytes by hM4Di, CNO (1 mg/kg b.w.) was administered 30 min before the training, and the LC neurons sending inputs to ACC were activated during the training by blue light pulse trains of 10 ms and 20 Hz frequency.

#### Light delivery protocols

In experimental groups requiring light delivery during training or before testing days, the rats were first habituated to handling once on the first day before experimentation or testing. Each rat underwent CPA behavioural study in which they were kept in one conditioning compartment in the morning with no stimulus for 45 min. In the afternoon, in the other compartment, each rat from different groups either received blue light (473 nm) or yellow light (589 nm) depending upon the activation and inhibition of particular cells and paired with CRD conditioning. The rats received light (3 min ON followed by 3 min OFF) during 45 min of conditioning, with the last 5 min light off through a fibre optic cable connected to a 1 × 2 intensity division fibre-optic rotary joint (Doric Lenses Inc., Canada). In experiments involving light stimulation or inhibition on each testing day, the same light delivery protocol was performed for 20 min before the start of testing.

For the inhibition of LC neurons, rats received bilateral yellow light pulse trains of 15 ms of 20 Hz frequency, and power density ranges from 10.0 to 23.7 mW/mm^2^^48,81^. For the optogenetic activation of LC neurons, the rats were administered blue light pulse trains of 10 ms and 20 Hz frequency, and light intensity ranges from 8.5 to 16.5 mW/mm^2^^48^.

The optical stimulation of ACC astrocytes or ACC astrocytic β2AR was performed using following parameters, 45 ms light pulse of 20 Hz frequency and 50 ms light pulse of 10 Hz frequency, were used, respectively^42,72^ with light intensity 10–15 mW/mm^2^. All these parameters were programmed using a waveform generator (Model AFG2021-SC; Tektronix) that is connected to either a blue or yellow laser source depending on the nature of the experiment (CNI Laser, China). The light stimulation protocols were consistent throughout all the experiments.

#### Cell-specific knockdown of β2AR receptors

We performed genetic knockdown of either astrocyte-specific β2AR receptors or neuron-specific β2AR receptors in ACC using a microRNA-based silencing technique. To achieve the knockdown efficiency, the BLOCK-iT Pol II miRNAi expression vector kits were used (Invitrogen). Six pre-miRNA sequences for β2AR receptors (β2AR receptors-miRNA) and a negative control sequence (NC-miRNA) were designed using Invitrogen’s RNAi Designer, created, and cloned into a pAAV-CMV-bGI-mCherry-miRNAi vector (Taitool Bioscience, China). The knockdown efficiency was then assessed by co-transfecting EGFP-tagged β2AR receptors with the β2AR receptors miRNA vectors in human embryonic kidney (HEK293) cell line. The knockdown efficiency was confirmed by decrease in the fluorescence signal expressed by the EGFP-β2AR receptors vector. The sequence with higher knockdown efficiency was chosen as follows: β2AR receptors -miRNA, TGCTGTCGTGAAGAAGTCACAGCAAGGTTTTGGCCA-CTGACTGACCTTGCTGTCTTCTTCACGA, and NC-miRNA, TGCTGTATAGGTCAAGTC-TAAGTCGAGTTTTGGCCACTGACTGACTCGACTTACTTGACCTATA. The selected oligos were then either cloned into the linearized pAAV-gfaABC1D-mCherry-miRNAi or pAAV-hSyn-mCherry-miRNAi vector (Taitool Bioscience, China) using T4 DNA ligase. The pAAV-gfaABC1D-mCherry and pAAV-hSyn-mCherry were used as control vectors, respectively. The plasmids were packaged into the AAV2/5 virus by calcium phosphate transfection with capsid and helper vectors on HEK293 cells. The pooled viruses were then purified by iodixanol density gradient centrifugation. After packaging, the AAV2/5 virus with gfaABC1D or hSyn promotor was used to knockdown the β2AR receptors either in the ACC astrocytes or neurons respectively. 0.8 µl of AAV2/5-gfaABC1D-mCherry-miRNAi (rβ2AR; Taitool Bioscience, China, 1.58E + 13 vg/mL, diluted 1:10 in sterile PBS) was injected in one group of rats, and AAV2/5-hSyn-mCherry-miRNAi (rβ2AR; Taitool Bioscience, China, 1.00E + 13 vg/mL, diluted 1:10 in sterile PBS) in other group of rats was injected bilaterally into ACC respectively. The sham rats in each group received same volume of either AAV2/5-gfaABC1D-mCherry-miRNAi (NC)-WPRE-pA (Taitool Bioscience, China, 1.58E + 13 vg/mL, diluted 1:10 in sterile PBS) and/or AAV2/5-hSyn-mCherry-miRNAi (NC)-WPRE-pA (Taitool Bioscience, China, 1.13E + 13 vg/mL, diluted 1:10 in sterile PBS) into ACC. After three weeks of virus injection, some rats from both groups were sacrificed and brains were collected to validate the knockdown of β2AR in the ACC through immunostaining and western blotting. At the same time, the rest of the rats were used for the behavioural experiment to assess the knockdown effect of β2AR on aversive learning and memory.

#### Chemogenetic manipulation

The rats were anesthetized with general anaesthetic sodium pentobarbital at the dose rate of 40 mg/kg of the body weight and placed on a stereotaxic apparatus. The skull was exposed via incision and 700 nl of diluted (1:10 in sterile PBS) virus construct AAV5-GFAP-hM4D(Gi)-mCherry (titer: 1.0 × 10^13^ genome copies per ml; Addgene, 50479-AAV5) was injected bilaterally into the ACC. The injection volume and flow rate (0.1 ml/min) was controlled by an injection pump (World Precision Instruments, USA). The rats were kept undisturbed for three weeks after virus injection to recover and allow gene expression. After  three weeks, these SD rats were divided into two groups; viz CNO injected group to activate the astrocytic hM4D(Gi) receptors and the saline-injected group as controls. In a separate group of rats to observe the effect of CNO alone during conditioning on CPA score and c-Fos expression, rats were infused with the same volume of the control construct AAV5-GFAP-mCherry into ACC. Clozapine-N-oxide hydrochloride (CNO; Sigma) was dissolved in dimethyl sulfoxide (DMSO) and then diluted in 0.9% saline giving a final DMSO concentration of 0.5%. The same concentration of DMSO prepared in saline solution was used for control^30^. To activate the hM4D(Gi) receptors, 1 mg/kg of CNO was injected intraperitoneally 30 min either before the start of training or before the testing days. The chosen dose of CNO does not produce any behavioural signs of seizure activity.

#### Behavioural assays

Rats from different experimental groups were randomly allocated to cages, and the experimenter was blind to treatment groups. All experiments were performed under the same experimental conditions to prevent any bias arising from the experiment. The number of animals has been specified in the figure legends.

##### Conditioned place avoidance paradigm

We measured an aversive learned behaviour that directly reflects the affective component of visceral pain, when a visceral pain assay combines the colorectal distension (CRD) with the conditioned place avoidance environment (CPA)^15,16^. The task comprised of a pre-conditioning day (day 1), conditioning days (days 2–5), and post-conditioning days (total 7 test days). When CRD was paired with a specific environmental context on the post-conditioning test days, the rats spent considerably less time in this compartment as compared to the pre-conditioning day. Our previous observation and current data showed that no anxiogenic behaviour and no significant differences in locomotor behaviour were observed during conditioning days. The detailed experimental protocols have been well described in our previous publications^8,15,16^. Briefly, the apparatus consisted of three wooden chambers (45 × 45 cm each). Two chambers were conditioning rooms with any of the room was paired with subthreshold pressure (<35 mm Hg) of CRD, and the third was a neutral chamber. Three chambers were categorized according to distinct visual and olfactory cues. The horizontal one had black horizontal stripes on the walls and an odour of 1.0% acetic acid. The vertical conditioning compartment had vertical streaks and was associated with a cinnamon scent. The walls with uniform appearance and no distinctive odour formed the neutral chamber. The floors of the conditioning chambers were characterized with tactile wooden coverings. The neutral chamber contained two doors, with an opening to each conditioning chamber. During conditioning, the doors could be closed to constrain the animal within a single conditioning chamber. The entrance of each compartment was opened on day 1, and each rat was given free access to explore the entire apparatus (all three chambers) for 20 min. The amount of time spent by each rat in each chamber was noted. On pre-conditioning day, no particular preferences for any of the chambers were detected, indicating that rats did not prefer any chamber over the other before conditioning. The animals disqualified the selection criteria and are excluded from further study if they spent less than 4 min or more than 16 min in any of the conditioning compartments. None of the rats meet this criterion, and none were excluded from the studies. The conditioning phase consisted of 4 consecutive days. In the morning, rats received no treatment, and were randomly confined to one of the conditioning chambers for 45 min. In the afternoon, rats received treatment (drug infusion, chemogenetic, optogenetic activation and/or inhibition, or knockdown of a particular cell) paired with subthreshold pressure (<35 mm Hg) of CRD in the other conditioning chamber for 45 min. CRD (<35 mm Hg) was produced by rapidly injecting saline into the colonic balloon and maintaining the distension for 30 s, repeated 5 times with 3 min intervals. The pressure was regulated with a pressure monitor device (World Precision Instruments, USA). After the completion of conditioning phase, on test days, each rat was given free access to move freely through three chambers for 20 min with no noxious aversive stimulus (CRD). The time spent within each chamber was recorded. The time spent in the conditioning context (paired with CRD) on the post-conditioning test days was subtracted from the time spent in the same compartment on the pre-conditioning day.

##### Visceromotor response to colorectal distension

The studies of VMRs to graded pressures of CRD were performed on rats to determine the effects of immunotoxic depletion of LC noradrenergic neurons, optogenetic activation of LC neurons, optical stimulation of ACC astrocytic β2ARs, knockdown of ACC astrocytic β2ARs and Gi activation of ACC astrocytes on visceral pain sensation. Briefly, 32-gauge Teflon coated stainless steel wires were implanted in the external oblique pelvic muscles after which the rats were given one week to recover from surgery. Graded-pressure of CRD (20, 40, and 60 mm Hg) was produced by rapidly injecting saline into a balloon over one second and maintaining the distention for 30 s. The simulation data was extracted using MATLAB MathWorks, and the results of muscle contraction (electromyography) were computed by determining the area under the curve (AUC), which is the sum of all recorded data points divided by the sample interval (in seconds) after baseline subtraction^6,46^.

##### Morris water maze

We also used the Morris water maze (MWM) to see the knockdown effect of ACC astrocytic β2ARs on spatial learning and memory in rats. The experiment was performed in a black circular tank^82^. The tank had a diameter of 150 cm and with a depth of 60 cm; it was filled with the water (22–24 °C) and made non-transparent by adding non-toxic black dye. A circular platform with a diameter of 10 cm and 25 cm high was lowered in the centre of the target quadrant 2.0 cm below water level. The animal behavioural data were acquired with the help of a digital video camera located above the centre of the tank and connected to ANY-maze software (Stoelting Co., USA). To assess the spatial learning in the rats, the training was performed for four consecutive days. During each trial, rats were released from any of the four different randomized visual location points/cues facing the tank wall and the curtains around the tank to learn and locate the hidden platform in a 60 s trial. The rats locate the platform by themselves, or they were driven by the experimenter to find the platform and then remained on the platform for 10 s. The three trials with an interval of 30 min between trials were conducted each day. To evaluate spatial learning and memory, a single probe test 24 h or 30 days after the last training session was performed. During the probe tests, the hidden platform was removed, and rats were released from the furthest location of the original platform. Then they were allowed to find the target quadrant that previously contained the hidden platform to assess their memory retention for the hidden underwater platform. All the trials were recorded, and latency to reach and locate the platform (sec), distance travelled (m), swimming speed (m/second), and the total time spent in the target quadrant (% of total time) were calculated.

##### Open field test (OFT)

The open field test is often used to assess the exploratory behaviour in a novel environment and offers a preliminary screening test for anxiety-related behaviour in rats^83^. We performed the open field task in rats who underwent knockdown of ACC astrocytic β2ARs receptors to assess the locomotor activity and anxiety-like exploratory behaviour. Briefly, the animals were first habituated to the behavioural testing room for 1–2 h on two consecutive days. Then the rats were placed individually in the centre of a 40 × 40 cm square sketched in the centre of a black square apparatus (80 × 80 × 40 cm). They were then allowed to explore the field freely for 15 min. The apparatus was thoroughly cleaned with 75% ethanol during each test. During spontaneous exploration behaviour in the open field, total horizontal distance travelled (m), number of rearings (times), time spent (s), and number of entries (times) in the centre were observed and recorded by ANY-maze (Stoelting Co., USA).

#### Immunohistochemistry and histological staining

After completing experiments, rats were anesthetized with urethane (1.5 g/kg) and transcardially perfused with 1×PBS, pH 7.4, followed by 4% paraformaldehyde in PBS. The brains were removed, placed in same fixative overnight at 4 °C, and cryoprotected in 30% sucrose solution for an additional 3 to 4 days at 4 °C. Each brain was then sectioned at 40 μm on a cryostat (Leica CM3050S Cryostat) and collected in cold 0.1% PBS.

For DβH immunoreactivity experiments, brain sections from vehicle and anti-DβH saporin treated rats were washed in 0.1% PBST and incubated in a 3% hydrogen peroxide solution for 30 min at room temperature. After one-time wash in 0.1% PBST, sections were incubated in blocking solution for one hour at room temperature. Sections were incubated in rabbit anti-DβH (1:1500, Abcam, Cat. No. ab96615) in 10% blocking solution at 4 °C for overnight. After 3 × 10 min washes in 0.1% PBST, next sections were incubated in biotinylated goat anti-rabbit IgG secondary antibody (1:500, Vector Laboratories, #BA-1000) 1 hour at room temperature. Then the sections were washed for 3 × 10 min in 0.1% PBST and incubated in an avidin-biotin ABC peroxidase solution (Vector Laboratories, #PK-6100) for 30 min at room temperature. Finally, the sections were washed three times in 1×PBS and stained using 3-3′diaminobenzidine-4 HCl (DAB) and nickel solution (Vector Laboratories, SK-4100) to produce a black staining product. Quantification of DβH immunoreactivity was visualized and performed on adjacent sections in locus coeruleus and sub-coeruleus region from approximate bregma −9.40 to −10.00^55^. All the analyses of the stained sections were performed by an observer blind to the experiment. The large images were scanned and stitched by Nikon Eclipse Ni-E upright fluorescence microscope for showing whole-brain section photo at 10× magnification. Then using a 100× oil objective, unambiguously positive cells were identified and counted.

For the c-fos experiments, rats were euthanized on the last day of conditioning at 90 min following the onset of either conditioning alone or conditioning with photo-stimulation or photoinhibition of LC neurons. To quantify the level of c-Fos in LC or ACC where appropriate, all images were taken under same experimental conditions, and individual maximum z-projections images were stitched together to produce an entire region. The images were then manually analyzed by an observer who was blind to the treatment condition with the help of ImageJ software, NIH USA. The number of DAPI positive cells in each image was marked and calculated. Relatively bright small uniform DAPI stained nuclei were not included in the counting process.

For all other immunostaining procedures, the rats were euthanized after the completion of the behavioural study and perfused with ice-cold PBS followed by 4% paraformaldehyde (PFA). Then brain samples were incubated in PFA overnight and transferred to 30% sucrose solution for three days. Brains were cryosectioned (Lecia CM3050 S Cryostat) at a thickness of 40 μm (coordinates 3.8 to 2.2 mm from bregma in case of ACC and −9.40 to −10.00 in case of LC). Then the sections were blocked by 10% normal goat serum in PBS with 0.3% Triton X-100 for 2 h and incubated with primary antibodies to mouse anti-GFAP (1:500, Sigma-Aldrich, Cat. No. G3893), rabbit anti-GFAP (1:500, Abcam, Cat. No. ab7260), rabbit anti-S100β (1:500, Abcam, Cat. No. ab41548), mouse anti-NeuN (1:500, Millipore, Cat. No. MAB-377), mouse anti-tyrosine hydroxylase (1:2000, Millipore, Cat. No. MAB-318), rabbit anti-Iba1 (1:500, Abcam, Cat. No. 178846), mouse anti-Iba1 (1:500, Abcam, Cat. No. 283319), chicken anti-GFP (1:500, Abcam, Cat. No. ab13970), rabbit anti-β1AR (1:500, Almone Labs, Cat. No. AAR-023), rabbit anti-β2AR (1:500, ThermoScientific, Cat. No. PA5 86339), mouse anti-β2AR (1:500, Santa Cruz, Cat. No. sc271322) and rabbit anti-c-Fos (1:500, Synaptic Systems, Cat. No. 226017). The sections were then washed and labelled with an appropriate alexa fluor secondary antibody (1:500). Finally, sections where appropriate were incubated with DAPI (1:20,000; Sigma) and mounted onto microscope slides, and covered with coverslips along with a fluorescent mounting medium (DAKO). The sections were photographed using a Zeiss Laser Scanning Microscope LSM 880. The quantitative images between comparative groups were acquired under 40× oil objective on the same day and under the same perimeters of the confocal microscope system setting to make the analysis comparable.

At the end of experiments that involve the infusion of the drug through a cannula, cresyl violet staining (Nissl staining) was used to mark the exact location of cannula placement into ACC and injection or optic fibre site into LC (Supplementary Fig. 11a–c). For this, rats were perfused and fixed with 4% PFA and brain sections were stained with cresyl violet. To check the diffusion of drug into ACC region, the rats were injected with 0.4 µl of 1% Chicago Sky Blue per hemisphere and euthanized 1 h after the injection (Supplementary Fig. 11d). The large images were scanned and stitched by Nikon Eclipse Ni-E upright fluorescence microscope for showing whole-brain section photo.

#### Western blot analysis

The sample preparation was carried out according to our previous publication^32^. Briefly, to measure the expression of learning-dependent genes, ACC was rapidly dissected at 90 min after the CPA training (final day of conditioning) or testing day 1 using an anodized aluminum brain slicer (Braintree scientific instruments) followed by homogenization in buffer containing 10 mM HEPES, 1 mM EDTA, 2 mM EGTA, 0.5 mM DTT, phosphatase, and protease inhibitor cocktails (Sigma-Aldrich). Homogenates were then centrifuged at 10,000 × *g* for 30 min at 4 °C. The supernatant was collected and considered as total protein for western immunoblot analysis. The total protein content of each brain homogenate fraction and synaptoneurosome fraction was determined using the Pierce^TM^ BCA protein assay Kit (ThermoScientific, USA). Forty micrograms of whole protein extract/lane were resolved using 12% SDS-PAGE and analyzed by western blot. The membranes were blocked in 5% non-fat milk in TBST buffer (containing 0.1% Tween 20) and incubated overnight at 4 °C. The following primary antibodies were diluted in block buffer: rabbit anti-pCREB (1:1000; Millipore, Cat. No. 06-519), rabbit anti-CREB (1:1000; Millipore, Billerica, MA, Cat. No.04-767), rabbit anti-ERK1/2 (1:1000; Cell Signalling Technology, Cat. No. 197G2), rabbit anti-β1AR (1:200, Almone Labs, Cat. No. AAR-023) and rabbit anti-β2AR (1:1000, ThermoScientific, Cat. No. PA5 86339) were used. α-tubulin (1:5000; Sigma-Aldrich, Cat. No. T6074) was used for loading normalization. The membranes were washed three times for 10 min each in TBST and then incubated with horseradish peroxidase-coupled specific secondary antibodies goat anti-rabbit IgG and goat anti-mouse IgG where appropriate (1:4000; Invitrogen) in TBS containing 5% skim milk for 1 h at room temperature. Western blots were visualized using the Immobilon® Western chemiluminescent HRP substrate (EMD Millipore Corporation, USA, catalogue No. WBKLS0500). Images were captured and processed by a gel documentation system (Azure Biosystems). Quantitative densitometric analysis was done using NIH ImageJ software. The band intensities obtained after densitometry were calculated as ratios of target antibody to tubulin. The results are expressed and presented in the respective figures as a percent of control samples mean values (for the quantification of phosphorylation proteins (pCREB and ERk1/2), both bands were averaged).

### Statistics and reproducibility

We used power analysis to estimate the sample size. For western blot studies, power calculation of one-way ANOVA predicted that four or five rats per group is necessary to gain the power of 0.8 and error probability of 0.05. A similar power analysis was calculated for behavioural experiments and indicated a minimum requirement of six sample size of each study for two-way ANOVA to achieve a power of 0.8 and an error probability of 0.05. The data are presented as mean ± SEM, and the statistical analysis where appropriate was carried out either using statistical package IBM SPSS statistics 20.0 (SPSS Inc, USA) or GraphPad Prism 9 (Graph Pad, CA). For the calculation of the CPA score, the amount of time spent in the conditioning compartment (paired with CRD) on the post-conditioning days (i.e., test days) was subtracted from the amount of time spent in the same box on the pre-conditioning day. These processes produced a CPA score for each rat. Then two-way ANOVA with repeated measures was applied to the CPA scores between different groups to see the significance of the data followed by multiple comparisons adjusted by Bonferroni’s multiple comparisons test. For western blot data analysis, one-way ANOVA followed by Tukey’s multiple comparison test was applied. The statistical analysis of the VMR data in different experimental groups was made by using two-way ANOVA, followed by multiple comparisons adjusted by the Bonferroni post hoc test. All the data collection and measurement were carried out on independent biological samples. When only two groups were compared, then for parametric distribution a two-tailed student t-test was and for non-parametric distribution two tailed Mann–Whitney test was applied.

### Reporting summary

Further information on research design is available in the Nature Portfolio Reporting Summary linked to this article.

## Supplementary information

Supplementary Information

Description of additional supplementary files

Supplementary Data 1

Supplementary Data 2

Reporting summary

## Data Availability

The data supporting the current study have not been deposited to any public repository but are available from the corresponding author on reasonable request. Source data used to generate bar figures are available as Supplementary Data 1. The uncropped and unprocessed blot images are provided as Supplementary Data 2.

## Code availability

This study did not generate code.
