# Supplementary material for: Adrenergic signalling to astrocytes in anterior cingulate cortex contributes to pain-related aversive memory in rats
Source: Commun Biol. 2023 Jan 5;6:10. doi: 10.1038/s42003-022-04405-6 (PMC9816175; doi:10.1038/s42003-022-04405-6)
Supplement: Supplementary file 1 — Supplementary Information [file 42003_2022_4405_MOESM1_ESM.pdf]

SUPPLEMENTARY MATERIAL

Adrenergic signaling to astrocytes in anterior cingulate cortex contributes to pain-related aversive memory in rats

Zafar Iqbal[1,2,3], Zhuogui Lei[1,2,3], Aruna S. Ramkrishnan[1,2], Shu Liu[1,2], Mahadi Hasan[1,2], Mastura Akter[1,2], Yuk Yan Lam[1,2,3] and Ying Li[1,2,3,4]*

[1]Department of Neuroscience, College of Veterinary Medicine and Life Sciences, City University of Hong Kong, Hong Kong

[2]Department of Biomedical Sciences, College of Veterinary Medicine and Life Sciences, City University of Hong Kong, Hong Kong

[3]Centre for Regenerative Medicine and Health, Hong Kong Institute of Science & Innovation, Chinese Academy of Sciences, Hong Kong SAR, P.R. China

[4]Centre for Biosystems, Neuroscience, and Nanotechnology, City University of Hong Kong, Hong Kong

***Corresponding aauthor email:** yingli@cityu.edu.hk

Table of Contents

Supplementary Fig. 1……………………………………………………………………3

Supplementary Fig. 2……………………………………………………………………5

Supplementary Fig. 3…………………………………………………………………....7

Supplementary Fig. 4……………………………………………………………………9

Supplementary Fig. 5……………………………………………………………………11

Supplementary Fig. 6……………………………………………………………………13

Supplementary Fig. 7……………………………………………………………………15

Supplementary Fig. 8…………………………………………………………………....17

Supplementary Fig. 9…………………………………………………………………....19

Supplementary Fig. 10…………………………………………………………………..21

Supplementary Fig. 11…………………………………………………………………..23

Supplementary Table 1, 2, 3, 4…………………………………………………………….25

Supplementary Table 5, 6, 7………………………………………………………………26

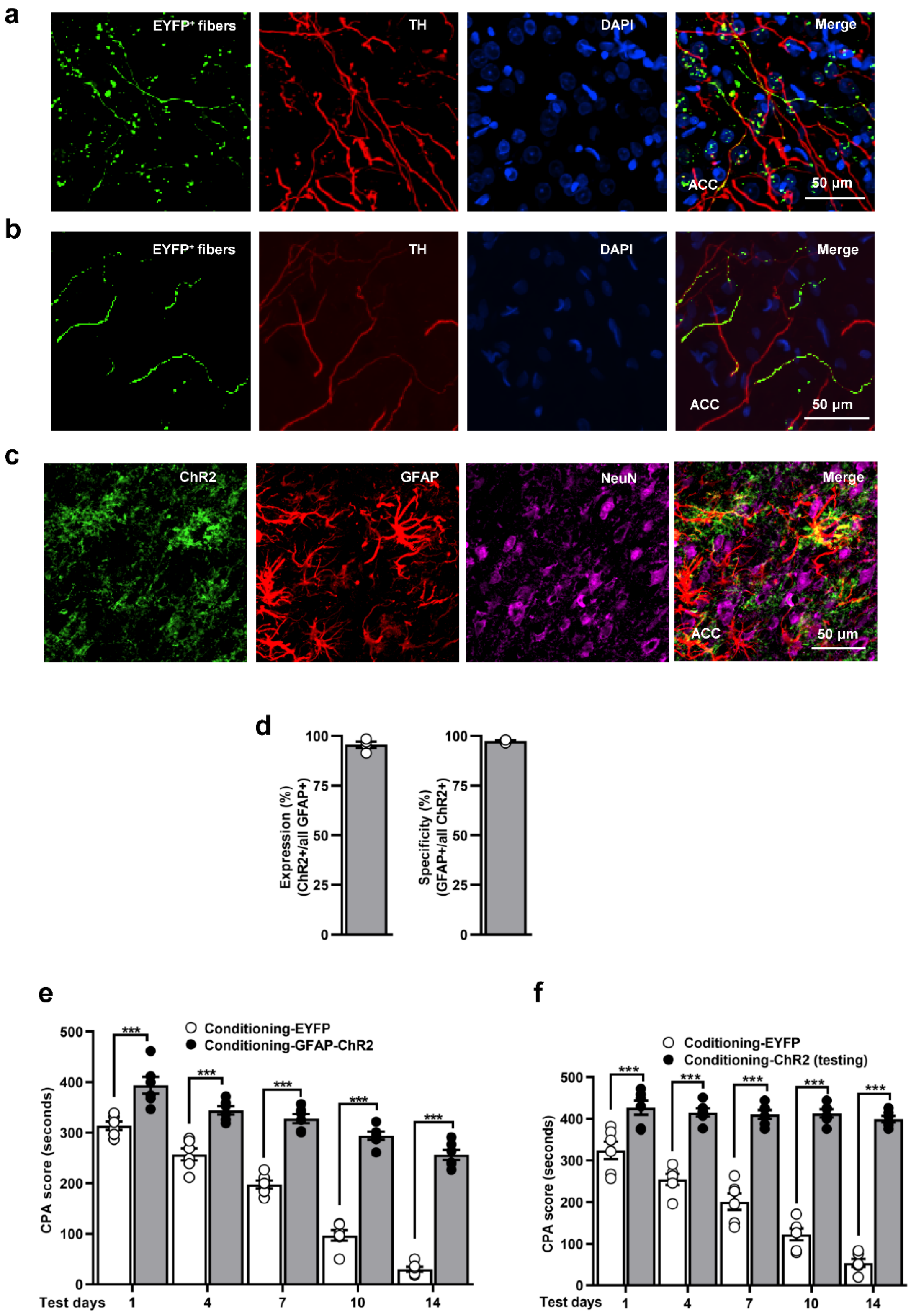
a
EYFP+ fibers
TH
DAPI
Merge
ACC
50 μm
b
EYFP+ fibers
TH
DAPI
Merge
ACC
50 μm
c
ChR2
GFAP
NeuN
Merge
ACC
50 μm
d
Expression (%) (ChR2+/all GFAP+)
Specificity (%) (GFAP+/all ChR2+)
e
Conditioning-EYFP
Conditioning-GFAP-ChR2
CPA score (seconds)
Test days
f
Coditioning-EYFP
Conditioning-ChR2 (testing)
CPA score (seconds)
Test days

Supplementary Figure 1. Effects of optogenetic manipulation of LC neurons on memory retrieval and ACC astrocytes on aversive memory formation. Related to Figure 2, 4 and 5.

(a) eNpHR3.0-$EYFP^+$ fibers co-stained with Th in the ACC region. Scale bar: 50 µm. **(b)** ChR2-$EYFP^+$ fibers co-stained with Th in the ACC region. Scale bar: 50 µm **(c)** Representative ACC astrocytes expressing ChR2 (green) with GFAP (red) and NeuN (Pink). Scale bar: 50µm. **(d)** Quantification of expression (%) and % specificity of GFAP-ChR2 in ACC astrocytes (n=4, three sections from each animal). **(e)** Optogenetic activation of ACC astrocytes significantly increased the CPA score compared to EYFP rats (blue light pulse 45 msec, 20Hz frequency, 3 minutes ON and 3 minutes OFF; n=6/group; ***p<0.0001, two-way ANOVA with Bonferroni test). **(f)** CPA score in EYFP and ChR2 rats when optogenetic stimulation of LC neurons was performed before testing days (n=6/group; ***p<0.0001, $F_{(4, 50)}$=24.07, two-way ANOVA with Bonferroni test. The results are presented as mean±SEM.

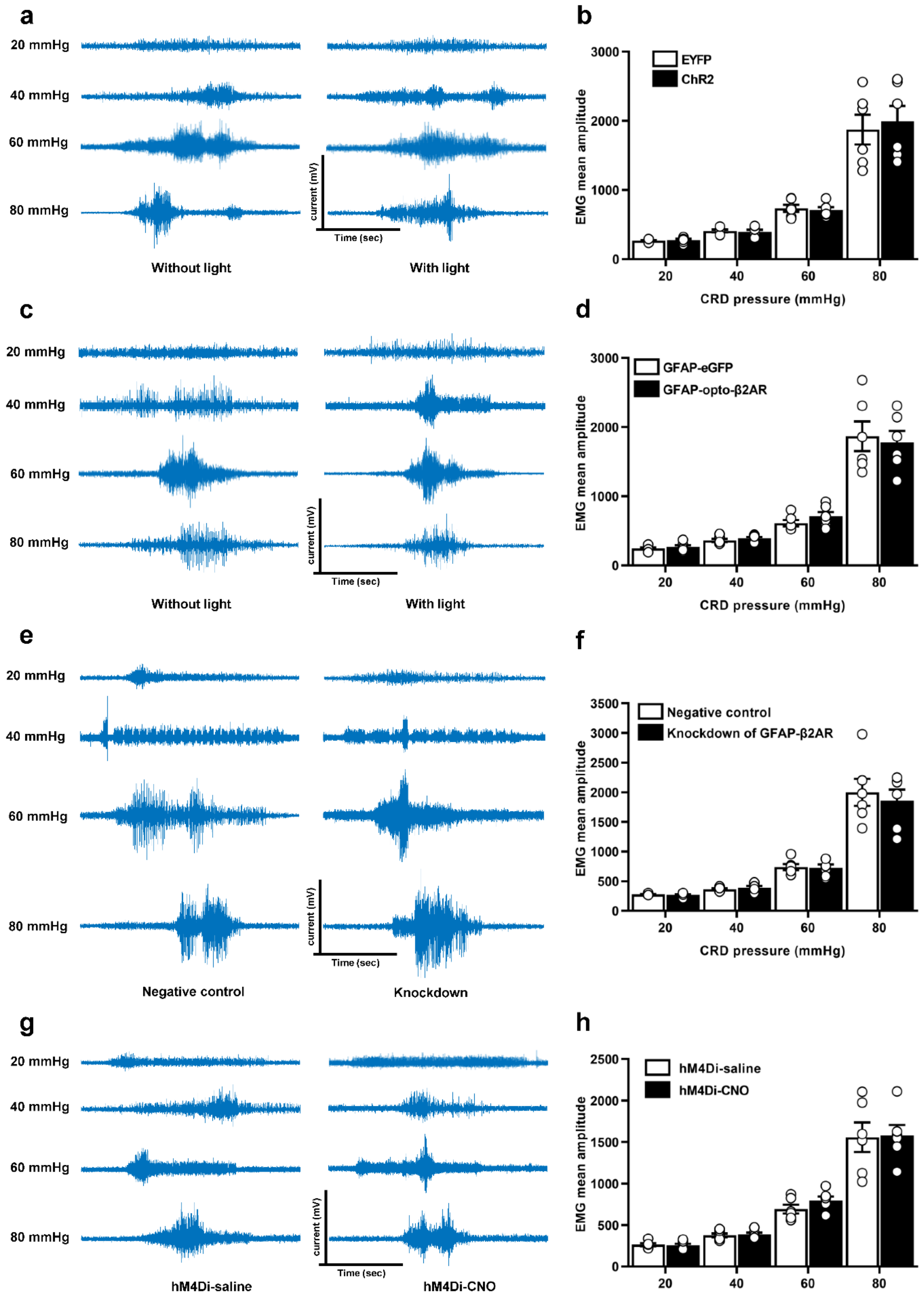
a
20 mmHg
40 mmHg
60 mmHg
80 mmHg
current (mV)
Time (sec)
Without light
With light
b
EYFP
ChR2
EMG mean amplitude
CRD pressure (mmHg)
c
Without light
With light
d
GFAP-eGFP
GFAP-opto-β2AR
e
Negative control
Knockdown
f
Negative control
Knockdown of GFAP-β2AR
g
hM4Di-saline
hM4Di-CNO
h
hM4Di-saline
hM4Di-CNO

Supplementary Figure 2. VMR recording analysis showing VMR response during cell-specific manipulations in LC and ACC regions. Related to Figures 4, 5, 6 and Supplementary Figure 9.

(a) Representative visceromotor response (VMR) recordings to graded pressures (20, 40, 60, 80 mm Hg) of colorectal distention (CRD) during optogenetic stimulation of LC neurons in EYFP and ChR2 rats. **(b)** The amplitude of electromyography (EMG) to CRD in control and light-activated LC neurons (n=6/group; p=0.9143, $F_{(3,40)}$=0.1726, two-way ANOVA). **(c)** Representative visceromotor response (VMR) recordings to graded pressures (20, 40, 60, 80 mm Hg) of CRD in eGFP and opto-β2ARs rats. **(d)** The amplitude of electromyography (EMG) to CRD in control and photoactivation of ACC astrocytic β2ARs (n=6/group; p=0.8304, $F_{(3,40)}$=0.2927, two-way ANOVA). **(e)** Representative visceromotor response (VMR) recordings to graded pressures (20, 40, 60, 80 mm Hg) of CRD in control and ACC astrocytic β2ARs depleted rats. **(f)** The amplitude of electromyography (EMG) to CRD in negative control and ACC astrocytic β2ARs knockdown rats (n=6/group; p=0.8836, $F_{(3, 40)}$=0.2176, two-way ANOVA). **(g)** VMR recordings to graded pressures (20, 40, 60, 80 mm Hg) of CRD in saline and CNO administered rats. **(h)** The amplitude of electromyography (EMG) to CRD in saline and CNO injected rats (n=6/group; p=0.9117, $F_{(3, 40)}$=0.1765, two-way ANOVA). These different cell-specific cellular manipulations in LC and ACC region have no effect on VMR response. All results are presented as mean±SEM.

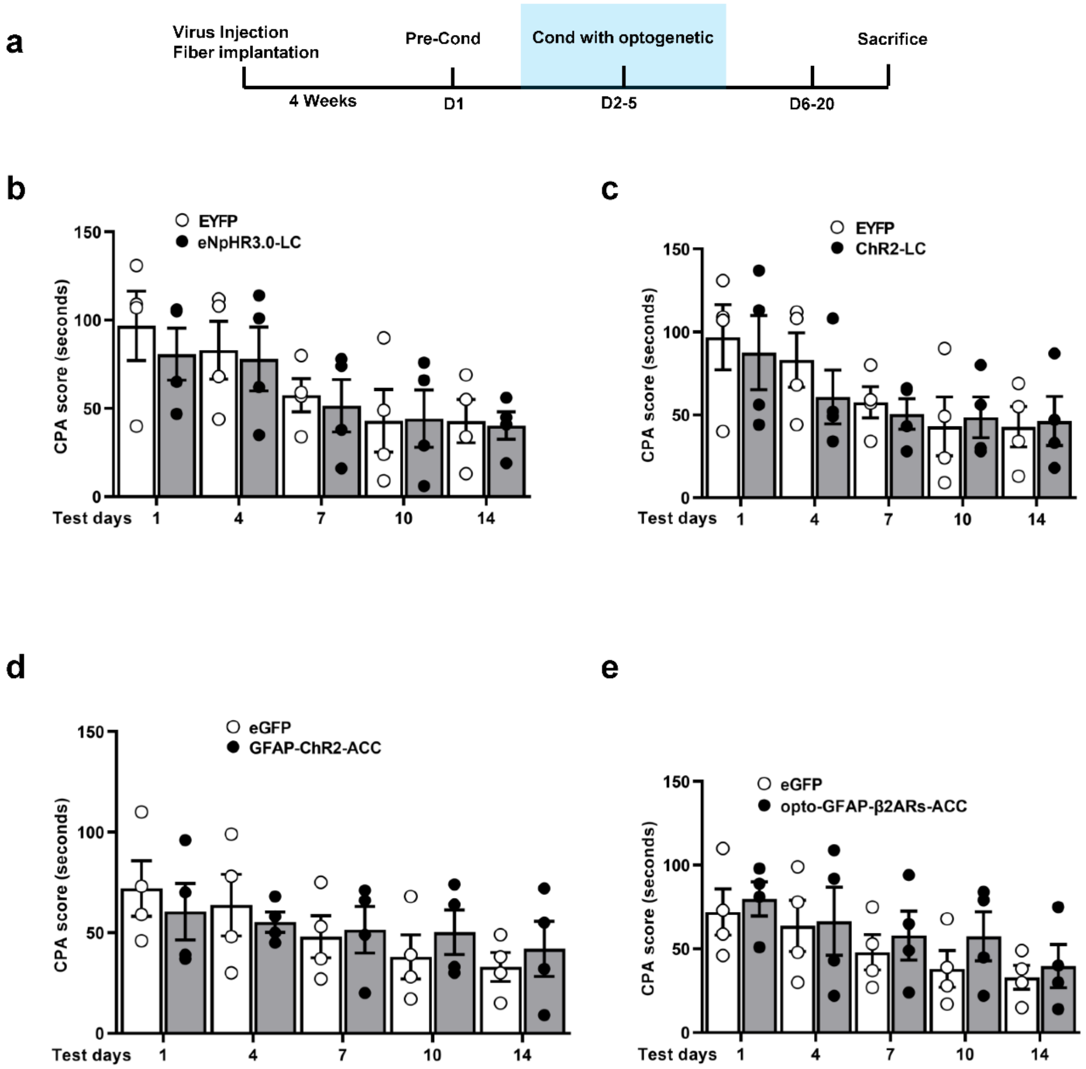
a
Virus Injection
Fiber implantation
Pre-Cond
Cond with optogenetic
Sacrifice
4 Weeks
D1
D2-5
D6-20
b
EYFP
eNpHR3.0-LC
CPA score (seconds)
Test days
c
EYFP
ChR2-LC
d
eGFP
GFAP-ChR2-ACC
e
eGFP
opto-GFAP-β2ARs-ACC
0
50
100
150
1
4
7
10
14

Supplementary Figure 3: Optogenetic manipulation of different cell types in the absence of CRD has no effect on aversion learning and memory formation, Related to Figures 2, 4, 5 and Supplementary Figure 1.

(a) Schematic shows the experimental timeline for optical manipulation of different cell types in LC and ACC region. **(b)** Effects of optical inhibition of LC neurons projecting to ACC on CPA Score. (n=4/group; p=0.9849, $F_{(4, 30)}$=0.08986, two-way ANOVA). **(c)** Effects of optical stimulation of LC neurons projecting to ACC on CPA score. (n=4/group; p=0.9032, $F_{(4, 30)}$=0.2568, two-way ANOVA). For panels B and C, same group of rats were used as control. **(d)** Effects of optical stimulation of ACC astrocyte on CPA Score. (n=4/group; p=0.8044, $F_{(4, 30)}$=0.4037, two-way ANOVA). **(e)** Effects of optical stimulation of ACC astrocytic β2AR on CPA Score. (n=4-5/group; p=0.9791, $F_{(4, 30)}$=0.1071, two-way ANOVA). For panels D and E, same group of rats were used as control. Results are presented as mean±SEM.

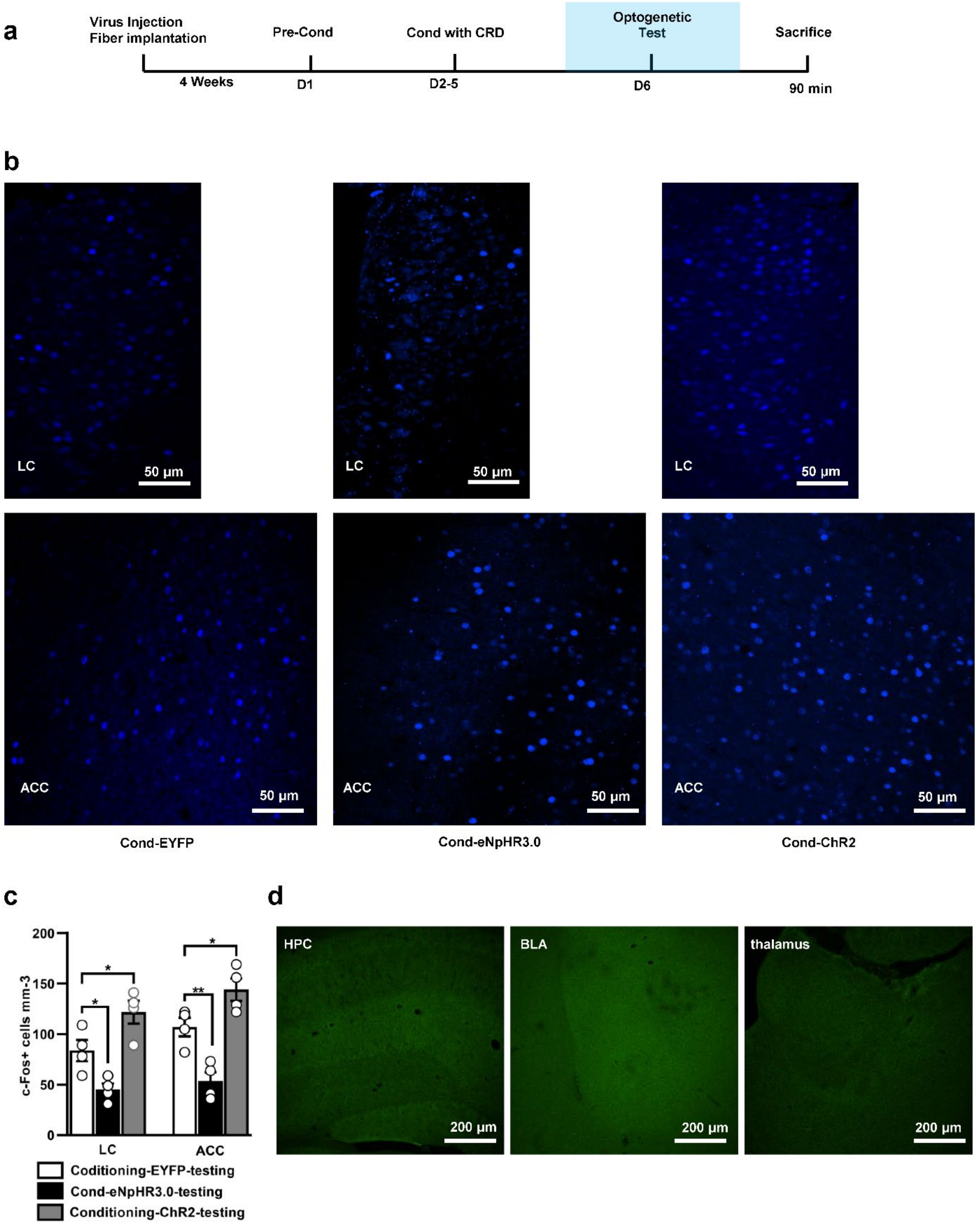
a
Virus Injection
Fiber implantation
Pre-Cond
Cond with CRD
Optogenetic
Test
Sacrifice
4 Weeks
D1
D2-5
D6
90 min
b
LC
50 µm
ACC
Cond-EYFP
Cond-eNpHR3.0
Cond-ChR2
c
c-Fos+ cells mm-3
200
150
100
50
0
LC
ACC
Coditioning-EYFP-testing
Cond-eNpHR3.0-testing
Conditioning-ChR2-testing
d
HPC
BLA
thalamus
200 µm

Supplementary Figure 4: Effects of optogenetic manipulation of LC neurons projecting to ACC before testing on c-Fos expression, Related to Figures 2 and 4.

(a) Schematic showing experimental timeline for optical inhibition and stimulation before testing day 1. **(b)** Representative images of c-Fos expression in LC and ACC of EYFP, eNpHR3.0 and ChR2 rats respectively. Scale bar: 50 µm. **(c)** Quantification of c-Fos^+ cells in the LC and ACC region after optogenetic inhibition (yellow light pulse 15 ms, 20Hz frequency, 3 minutes ON, 3 minutes OFF; *p=0.034(LC); **p=0.0069(ACC) and optogenetic activation of LC neurons projecting to ACC (blue light pulse 10 ms, 20Hz frequency, 3 minutes ON, 3 minutes OFF; n=4 rats/group, three sections from each animal; *p=0.036(LC); *p=0.045(ACC), one-way ANOVA). Results are presented as mean±SEM. **(d)** Representative images showing no retrograde expression of Cre-dependent eNpHR3.0 in hippocampus, amygdala and thalamus region. Scale bar: 200 µm.

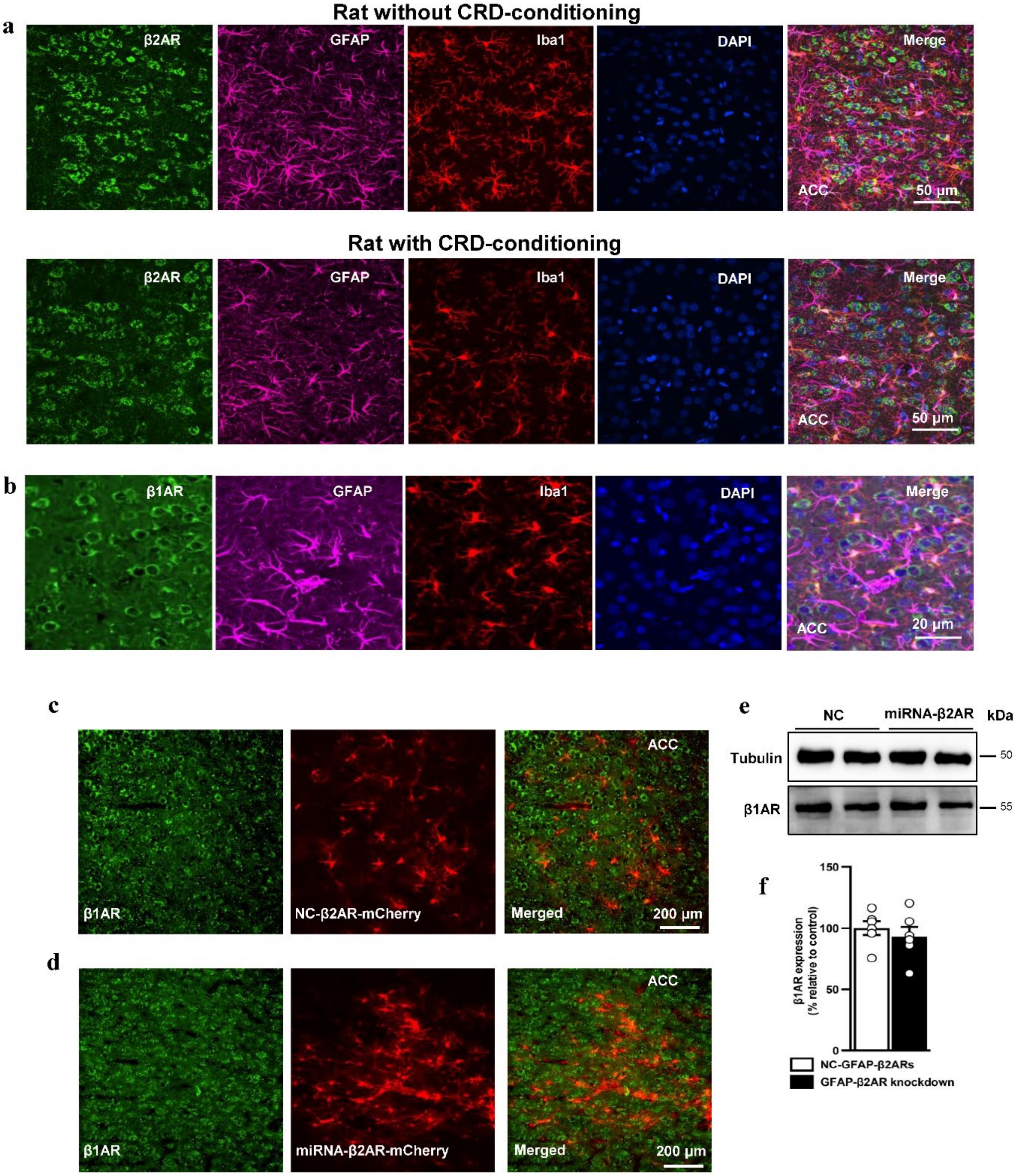
Rat without CRD-conditioning
a
β2AR
GFAP
Iba1
DAPI
Merge
ACC
50 μm
Rat with CRD-conditioning
β2AR
GFAP
Iba1
DAPI
Merge
ACC
50 μm
b
β1AR
GFAP
Iba1
DAPI
Merge
ACC
20 μm
c
ACC
β1AR
NC-β2AR-mCherry
Merged
200 μm
d
ACC
β1AR
miRNA-β2AR-mCherry
Merged
200 μm
e
NC
miRNA-β2AR
kDa
Tubulin
50
β1AR
55
f
β1AR expression
(% relative to control)
150
100
50
0
NC-GFAP-β2ARs
GFAP-β2AR knockdown

Supplementary Figure 5. Expression pattern of β2ARs, β1ARs in glial cells and knockdown effect of miRNAi(rβ2AR) on expression level of β1ARs in ACC astrocytes. Related to Figures 5 and 6.

(a) Co-staining of β2ARs with GFAP and Iba1(microglia marker) in the ACC region of unconditioned and conditioning rats respectively. Scale bar: 50 μm. **(b)** Expression of β1ARs in the ACC astrocytes of sham rats. Scale bar: 20 μm. **(c, d)** Representative images of negative control (c) and β2AR-mCherry virus (d; red) co-stained with ACC astrocytic β1ARs (green). Scale bar: 100 μm. **(e, f)** Representative blot images (e) and densitometric analysis (f) for β1AR expression following microinjection of negative control and mCherry-miRNAi(rβ2AR) virus into ACC (n=6/group; $p=0.5009$, $t_{10}=0.6983$, unpaired t-test). Results are presented as protein percentage of control sample mean values (100%). Protein values are normalized to those of tubulin. Results are expressed as mean ± SEM.

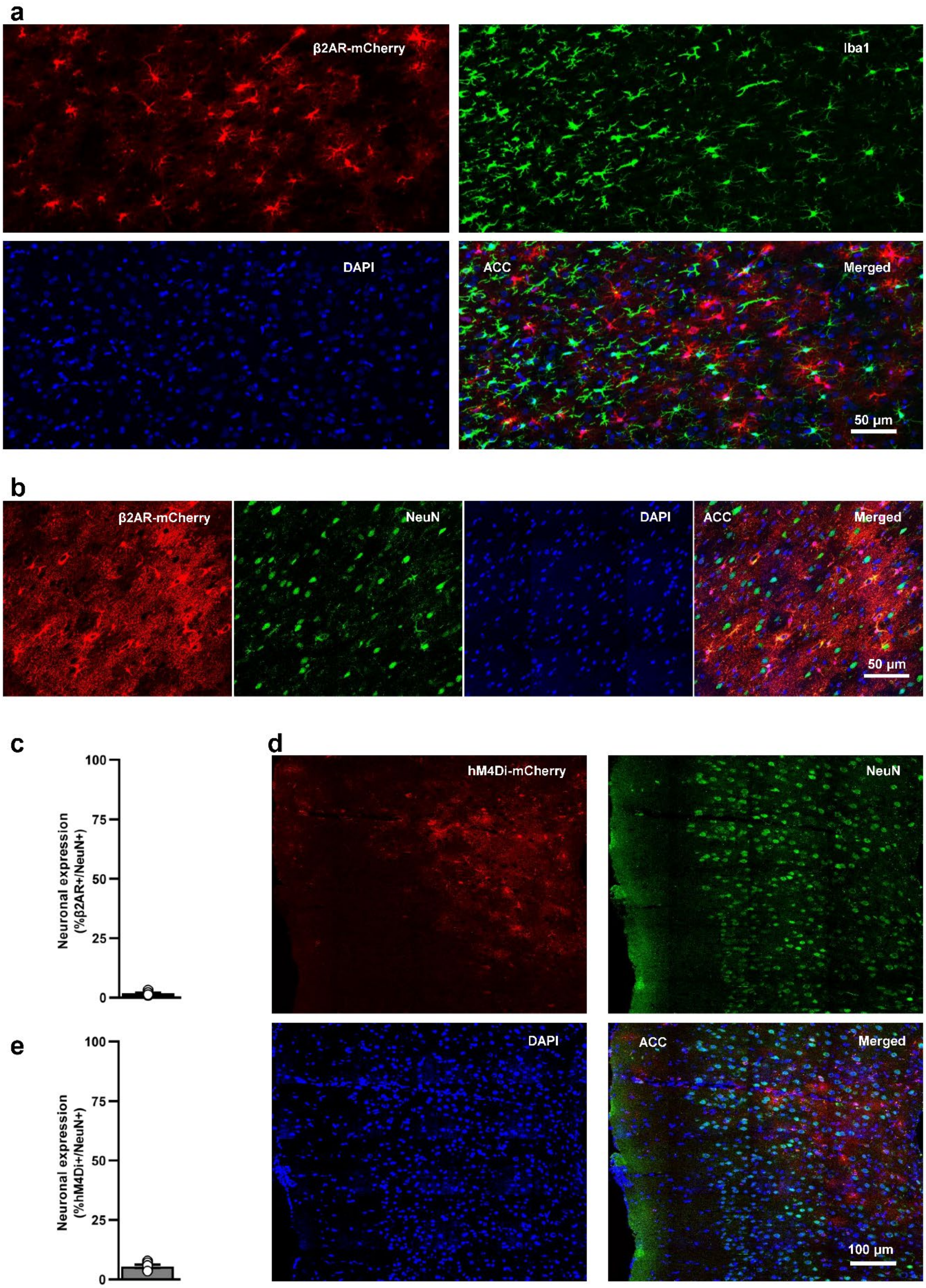
a
β2AR-mCherry
Iba1
DAPI
ACC
Merged
50 µm
b
β2AR-mCherry
NeuN
DAPI
ACC
Merged
50 µm
c
Neuronal expression (%β2AR+/NeuN+)
100
75
50
25
0
d
hM4Di-mCherry
NeuN
DAPI
ACC
Merged
100 µm
e
Neuronal expression (%hM4Di+/NeuN+)
100
75
50
25
0

Supplementary Figure 6. Off-target expression of cell-specific astrocytic promotor in microglia and neurons. Related to Figures 6 and Supplementary Figure 9.

(a) Representative images of AAV2/5-gfaABC1D-mCherry-miRNAi(rβ2AR) in the ACC region co-stained with Iba1 (a microglia activation marker). Scale bar: 50 µm. **(b)** Representative images of AAV2/5-gfaABC1D-mCherry-miRNAi(rβ2AR) in the ACC region co-stained with neuronal nuclear marker NeuN. Scale bar: 50 µm. **(c)** % expression of AAV2/5-gfaABC1D-mCherry-miRNAi(rβ2AR) in the ACC neurons (n=3, two sections from each animal). **(d)** Representative images of AAV5.GFAP.hM4Di.mCherry in the ACC stained with neuronal nuclear marker NeuN. Scale bar: 100 µm. **(e)** % expression of hM4Di-mCherry+ cells in ACC neurons (n=3, two sections from each animal). Values are presented as mean ± SEM.

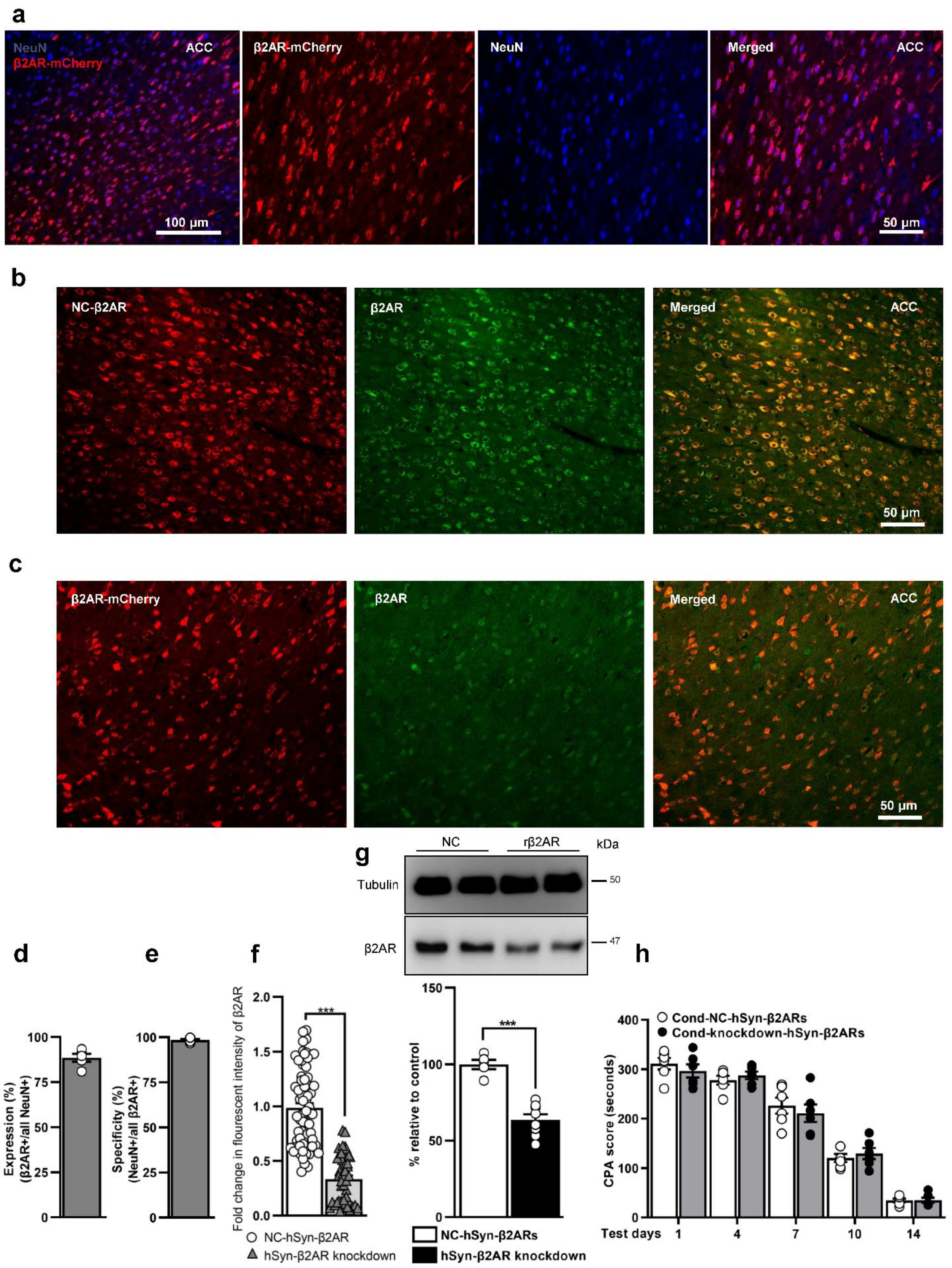
a
NeuN
β2AR-mCherry
ACC
100 μm
β2AR-mCherry
NeuN
Merged
ACC
50 μm
b
NC-β2AR
β2AR
Merged
ACC
50 μm
c
β2AR-mCherry
β2AR
Merged
ACC
50 μm
g
NC
rβ2AR
kDa
Tubulin
50
β2AR
47
% relative to control
NC-hSyn-β2ARs
hSyn-β2AR knockdown
d
Expression (%) (β2AR+/all NeuN+)
e
Specificity (%) (NeuN+/all β2AR+)
f
Fold change in flourescent intensity of β2AR
NC-hSyn-β2AR
hSyn-β2AR knockdown
h
Cond-NC-hSyn-β2ARs
Cond-knockdown-hSyn-β2ARs
CPA score (seconds)
Test days

Supplementary Figure 7. Cell-specific knockdown of β2ARs in ACC neurons did not contribute to visceral pain aversion learning and memory. Related to Figure 6.

(a) Representative images of virus construct: AAV2/5-hSyn-mCherry-miRNAi(rβ2AR) in the ACC region. β2AR-mCherry in ACC neurons (red) colocalized with neuronal nuclear marker NeuN. Scale bar: 100µm, 50µm. **(b)** Representative images of negative control (NC) virus in ACC neurons (red) colocalized with β2AR. Scale bar: 50µm. **(c)** Representative images of β2AR-mCherry with ACC neuronal β2AR. It indicates depletion of β2AR in ACC neurons. Scale bar: 50µm. **(d, e)** % expression (d) and specificity (e) of β2AR-mCherry virus in the ACC region respectively (n=5 rats/group, three sections from each animal). **(f)** Quantification of fold change in β2AR fluorescent intensity (n=4/group, three sections from each animal; ***p<0.0001, Mann Whitney test). **(g)** Representative western blot image and western blot analysis for β2AR expression following an injection of AAV2/5-hSyn-mCherry-miRNAi(rβ2AR) into ACC (n=5-8/group; ***p<0.0001, t_{11}=6.87, unpaired t-test). Results are presented as protein percentage of control sample mean values (100%). Protein values are normalized to those of tubulin. **(h)** The amount of CPA score in the NC and neuronal β2AR knockdown rats (n=6/group; p=0.6659, two-way ANOVA). All results are presented as mean±SEM.

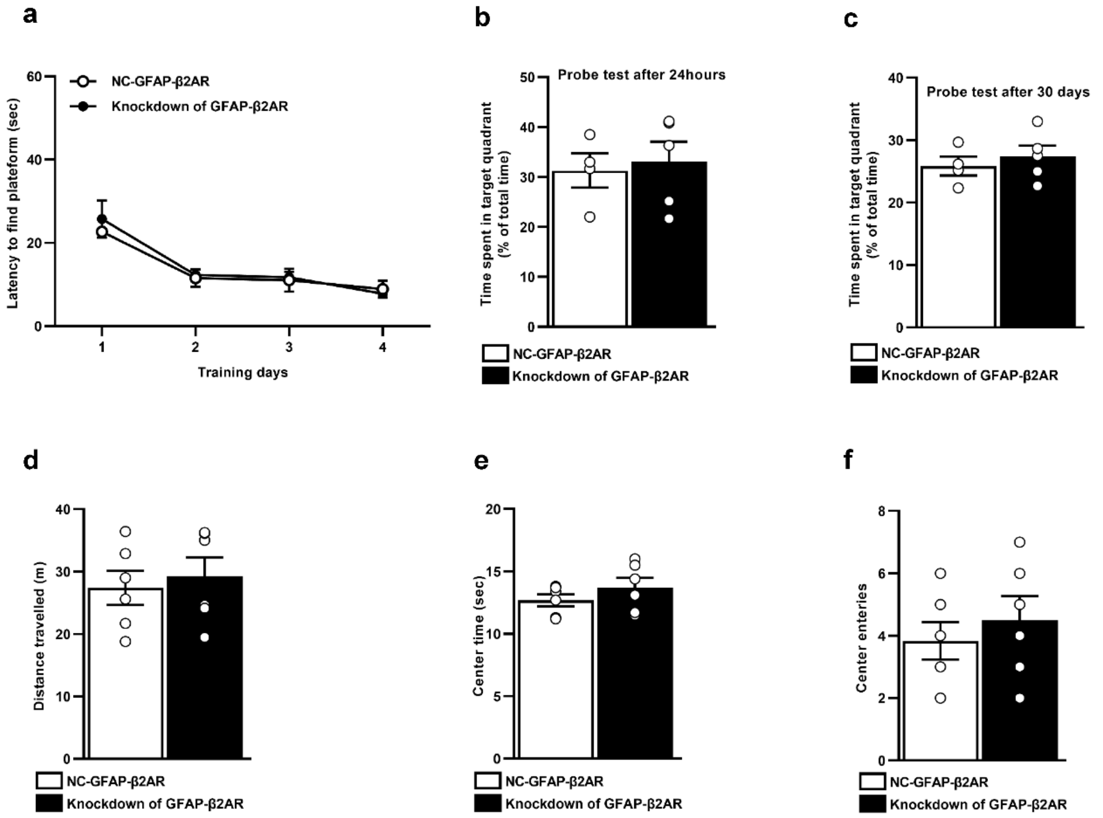
a
60
40
20
0
NC-GFAP-β2AR
Knockdown of GFAP-β2AR
Latency to find plateform (sec)
1
2
3
4
Training days
b
Probe test after 24hours
50
40
30
20
10
0
Time spent in target quadrant (% of total time)
NC-GFAP-β2AR
Knockdown of GFAP-β2AR
c
Probe test after 30 days
40
30
20
10
0
Time spent in target quadrant (% of total time)
NC-GFAP-β2AR
Knockdown of GFAP-β2AR
d
40
30
20
10
0
Distance travelled (m)
NC-GFAP-β2AR
Knockdown of GFAP-β2AR
e
20
15
10
5
0
Center time (sec)
NC-GFAP-β2AR
Knockdown of GFAP-β2AR
f
8
6
4
2
0
Center enteries
NC-GFAP-β2AR
Knockdown of GFAP-β2AR

Supplementary Figure 8. Effect of cell-specific knockdown of ACC astrocytic β2ARs on spatial reference memory and anxiety-like behavior. Related to Figure 6.

(a) Knockdown of β2AR in ACC astrocytes during the water maze task did not change the spatial learning and memory in rats. During the training sessions, the acquisition curve of rats to find the hidden platform from both groups showed no significant difference (n=4-5/group; p=0.8805, $F_{(3, 24)}$=0.2216, two-way ANOVA). **(b, c)** During a recent and remote spatial reference memory test in a 60 seconds probe test, the rats from both groups did not show any significant difference in the amount of time spent in the target location previously containing platform when tested after 24 hours (b; p=0.7607, t_7=0.3167, unpaired t-test) and 30 days of the trail respectively (c; p=0.5406, t_7=0.6432, unpaired t-test). **(d, e, f)** Anxiety-like behaviour in control and ACC astrocytic β2ARs depleted rats tested in the open field (n=6/group). There was no significant difference observed in (d) total horizontal distance travelled (p=0.6572, t_{10}=0.4573, unpaired t-test), (e) time spent in the center arena (p=0.2926, t_{10}=1.111, unpaired t-test), and (f) the number of entries into the center arena (p=0.5083, t_{10}=0.6860, unpaired t-test). All results are presented as mean±SEM.

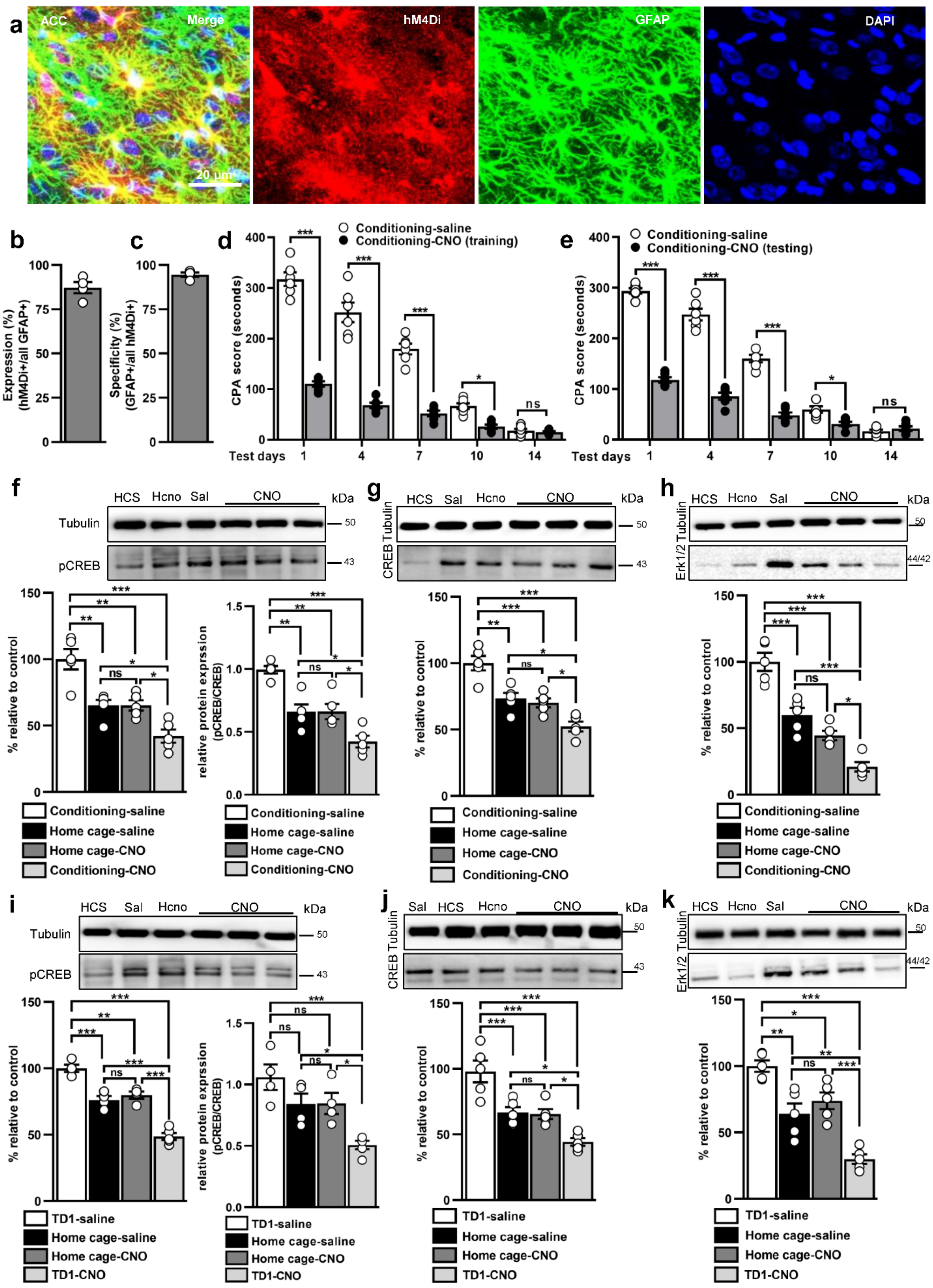
a
ACC
Merge
20 μm
hM4Di
GFAP
DAPI
b
Expression (%) (hM4Di+/all GFAP+)
c
Specificity (%) (GFAP+/all hM4Di+)
d
Conditioning-saline
Conditioning-CNO (training)
CPA score (seconds)
Test days
e
Conditioning-saline
Conditioning-CNO (testing)
f
HCS Hcno Sal CNO kDa
Tubulin
pCREB
% relative to control
relative protein exprssion (pCREB/CREB)
Conditioning-saline
Home cage-saline
Home cage-CNO
Conditioning-CNO
g
HCS Sal Hcno CNO kDa
Tubulin
CREB
h
HCS Hcno Sal CNO kDa
Tubulin
Erk1/2
44/42
i
HCS Sal Hcno CNO kDa
TD1-saline
Home cage-saline
Home cage-CNO
TD1-CNO
j
Sal HCS Hcno CNO kDa
k
HCS Hcno Sal CNO kDa

Supplementary Figure 9: Activation of Gi pathway in ACC astrocytes disrupts the acquisition and expression of aversion memory and learning-dependent molecular changes. Related to Figure 7.

(a) Representative images from rat expressing hM4Di-mCherrry in ACC astrocytes. Scale bar: 20µm. **(b, c)** % expression (b) and specificity (c) of hM4Di+ cells in GFAP+ cells of ACC region (n=4 rats/group, three sections from each animal). **(d, e)** CPA score in saline and CNO injected rats before training (d) or before testing days (e; n=6/group; ***p<0.0001, *p=0.01, ^{ns}p>0.9999 (training group); ^{ns}p=0.9860 (testing group); two-way ANOVA with Bonferroni test). **(f, g, h, i, j, k)** Representative images and averaged densitometric western blot analysis of pCREB, pCREB/CREB, CREB, and Erk1/2 expression measured either training (f, g, h) or testing day 1 (i, j, k) in saline, home cage-saline, home cage-CNO, and CNO administered rats (n=4-5/group; ***p<0.0001, **p<0.001, *p<0.01, ^{ns}p>0.9999 (f; pCREB), ^{ns}p>0.9999 (f; pCREB/CREB), ^{ns}p=0.9457 (g; CREB), ^{ns}p=0.1850 (h; Erk1/2), training group; ^{ns}p=0.8063 (i; pCREB), ^{ns}p=0.9999, 0.2616, 0.2772 (i; pCREB/CREB), ^{ns}p=0.9985(j; CREB), ^{ns}p=0.6306 (k; Erk1/2), testing group; one-way ANOVA with Tukey's test). Results are presented as protein percentage of control sample mean values (100%). Protein values are normalized to those of tubulin. All results are presented as mean±SEM. ns=non-significant, p>0.05.

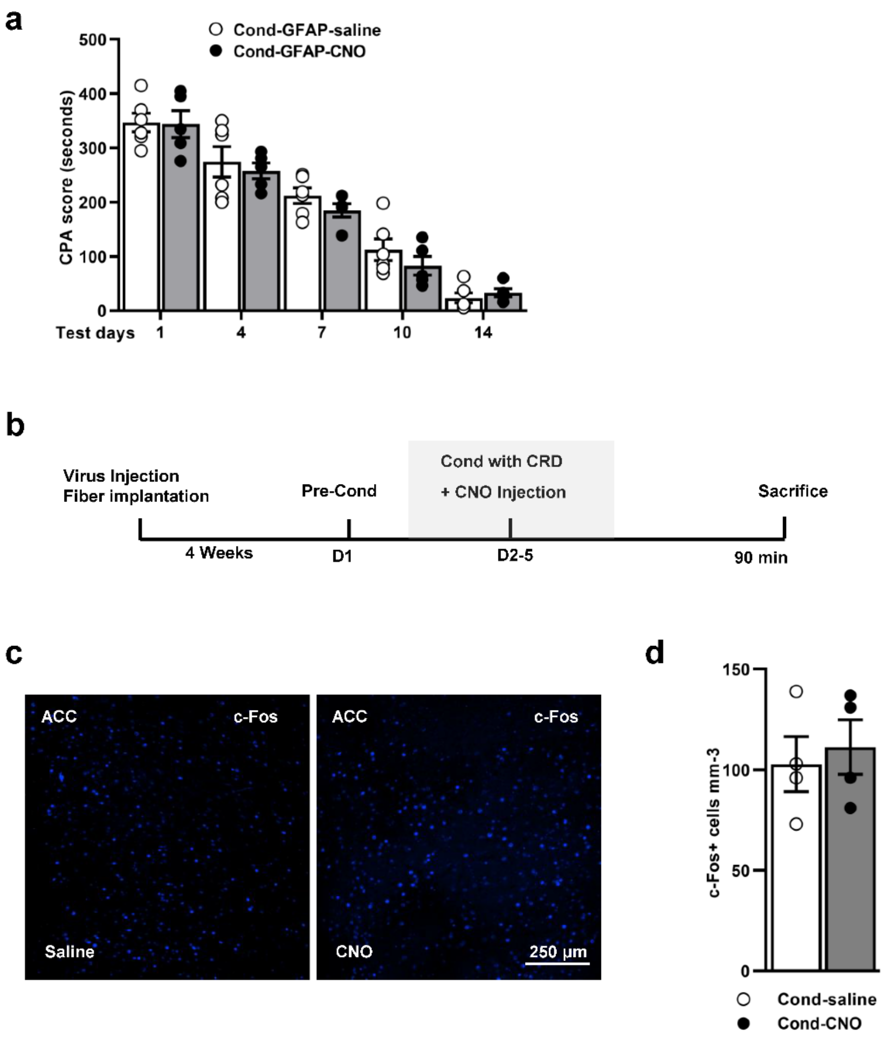
a
Cond-GFAP-saline
Cond-GFAP-CNO
CPA score (seconds)
500
400
300
200
100
0
Test days
1
4
7
10
14
b
Virus Injection
Fiber implantation
Pre-Cond
Cond with CRD
+ CNO Injection
Sacrifice
4 Weeks
D1
D2-5
90 min
c
ACC
c-Fos
Saline
ACC
c-Fos
CNO
250 μm
d
c-Fos+ cells mm-3
150
100
50
0
Cond-saline
Cond-CNO

Supplementary Figure 10: CNO application itself has no effect on CPA score and c-Fos expression, Related to Figure 7 and Supplementary Figure 9.

(a) CNO (1 mg/kg b.w) was administered to AAV5-GFAP-mCherry conditioned rats. CNO treatment alone does not affect the CPA score. (n=5-6/group; ***p=0.7841, $F_{(4, 45)}$=0.4329, two-way ANOVA). **(b)** Schematic showing experimental timeline. **(c)** Representative c-Fos images in the ACC region from saline and CNO infused conditioning rats. Scale bar: 250 µm. **(d)** CNO itself has no effect on c-Fos expression in saline and CNO administered conditioning rats (n=4/group with 3 sections from each rat; unpaired t-test). Results are presented as mean±SEM.

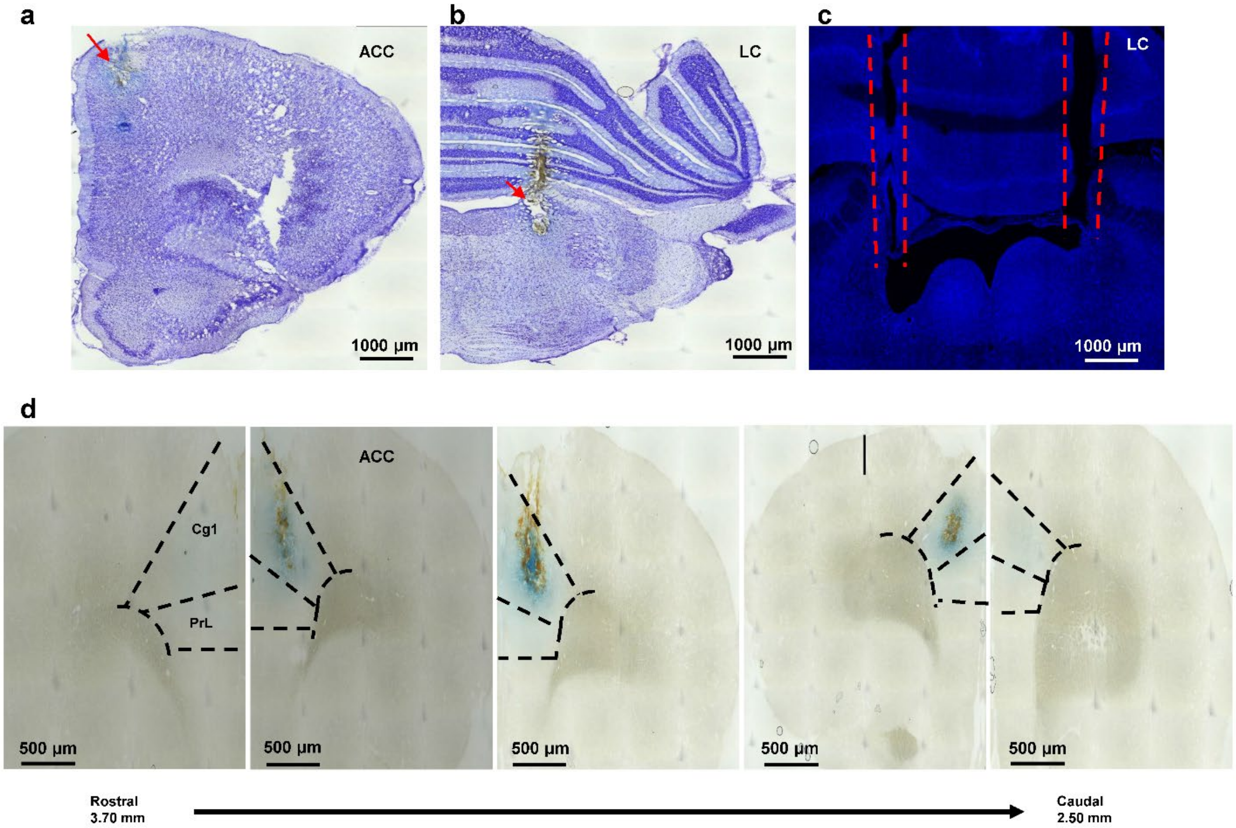
a
ACC
1000 µm
b
LC
1000 µm
c
LC
1000 µm
d
ACC
Cg1
PrL
500 µm
500 µm
500 µm
500 µm
500 µm
Rostral
3.70 mm
Caudal
2.50 mm

Supplementary Figure 11. Correct placement of chronic cannulation and fiber-optic implantation in ACC and locus coeruleus respectively and diffusion of Chicago Sky Blue after ACC injection. Related to Figures 1, 2, 3, 4, and 7.

(a) Representative cresyl violet-stained coronal section of rat brain shows the correct placement of bilateral chronic ACC cannula (red arrow) in the ACC region. Scale bar: 1000 µm. **(b, c)** Representative images of the brain showing microinjection sites (b; red arrow) and fiber implantation (c; red dotted lines) within locus coeruleus, respectively. Scale bar: 1000 µm. **(d)** Representative sections of the brain at 3.70 mm and 2.50 mm from bregma show diffusion of Chicago Sky Blue (1%) across the ACC region. Scale bars: 500 µm. Rats were injected bilaterally with 1µl of Chicago Sky Blue and sacrificed 1 hour after infusion. Cg1=cingulate cortex area 1, and Prl=prelimbic cortex

Supplementary Table 1: CPA score (mean±SEM) and statistical analysis for raw CPA score represented in Figure 1c and 1d respectively.

	Cond-vehicle		Cond-DβH saporin		Vehicle+U69593		DβH saporin +U69593	
Test days	Mean	SEM	Mean	SEM	Mean	SEM	Mean	SEM
1	303.33	9.36	100.5	4.36	280.33	14.21	242.16	10.80
4	274.66	11.05	68.33	6.45	162.50	21.26	137.16	14.88
7	200.33	13.21	43.50	4.18				
10	90.16	14.61	32	5.59				
14	33.50	6.49	18.00	5.96				

Statistics: $F_{(4,50)}$=47.90 (DβH saporin), p<0.0001; $F_{(1,20)}$=0.1659 (DβH saporin+U69593), p=0.6881; Cond=conditioning

Supplementary Table 2: CPA score (mean±SEM) and statistical analysis for raw CPA score represented in Figure 2f and 2g respectively.

	Training				Testing			
	Cond-EYFP		Cond-eNpHR3.0		Cond-EYFP		Cond-eNpHR3.0	
Test days	Mean	SEM	Mean	SEM	Mean	SEM	Mean	SEM
1	317.83	7.24	99	11.10	313.8	10.04	101.66	13.44
4	259.83	8.97	73.83	11.57	276.33	11.47	71.33	10.84
7	199	9.38	39.66	15.56	196.33	10.32	42.50	9.43
10	91.33	8.26	26.50	8.45	88.17	9.82	26.33	6.52
14	28.80	4.80	12.83	4.44	25.50	5.70	14	3.49

Statistics: $F_{(4,50)}$=40.37 (training), p<0.0001; $F_{(4,50)}$=43.55 (testing), p<0.0001; Cond=conditioning

Supplementary Table 3: CPA score (mean±SEM) and statistical analysis for raw CPA score represented in Figure 3a and 3b respectively.

	βAR antagonist				β2AR antagonist			
	Cond-saline		Cond-propranolol		Cond-saline		Cond-ICI118,551	
Test days	Mean	SEM	Mean	SEM	Mean	SEM	Mean	SEM
1	305.16	7.47	115.66	7.32	310.33	6.70	118.66	9.47
4	271.83	11.13	103.50	9.95	269.66	8.95	92.50	12.39
7	202.83	10.61	82	8.32	188	11.86	80	12.56
10	104.50	11.56	45.16	4.05	111.33	8.62	58	6.49
14	32.50	6.19	26.50	3.36	26.16	3.45	45	7.03

Statistics: $F_{(4,50)}$=40.64 (βAR antagonist), p<0.0001; $F_{(4,50)}$=45.41 (β2AR antagonist), p<0.0001; Cond=conditioning

Supplementary Table 4: CPA score (mean±SEM) and statistical analysis for raw CPA score represented in Figure 4f, 4g and 4h respectively.

	Cond-EYFP		Cond-ChR2		Cond-ChR2+saline-ACC		Cond-ChR2+prop-ACC		EYFP+U69593		ChR2+U69593	
Test days	Mean	SEM	Mean	SEM	Mean	SEM	Mean	SEM	Mean	SEM	Mean	SEM
1	300	12.20	417	13.60	410	6.64	137.50	6.02	249.33	15.89	222	12.79
4	246.83	15.68	391.66	3.95	380.83	7.65	118.83	7.65	160.33	12.74	143.83	8.27
7	187	13.48	375.66	8.26	370.16	9.19	89.60	5.15				
10	89.16	7.14	346	12.02	332.16	6.43	79.33	2.17				
14	17	2.89	319	8.58	316	8.71	72.50	6.16				

Statistics: $F_{(4,50)}$=26.34 (ChR2), p<0.0001; $F_{(4,50)}$=2.36 (ChR2-propranolol), p<0.01; $F_{(1,20)}$=0.1813 (ChR2+U69593), p=0. 6748; Cond=conditioning

Supplementary Table 5: CPA score (mean±SEM) and statistical analysis for raw CPA score represented in Supplementary Figure 1d, Figure 5d and 5e respectively.

	Cond-EYFP		Cond-GFAP::ChR2		Cond-eGFP		Cond-opto-β2AR		eGFP+U69593		opto-β2AR+U69593	
Test days	Mean	SEM	Mean	SEM	Mean	SEM	Mean	SEM	Mean	SEM	Mean	SEM
1	314	8.27	393.83	16.60	304	9.23	394	6.10	221	12.15	216.16	8.41
4	257	11.98	344.33	8.34	226.33	12.07	376.16	5.99	114.33	11.87	122.66	12.11
7	197.66	8.08	328.16	9.27	170.83	7.81	357.16	11.17				
10	97	10.41	294	8.14	94.83	7.66	334.1	4.59				
14	30	4.94	256.5	10.13	26	2.69	313.60	8.84				

Statistics: $F_{(4,50)}$=21.18 (GFAP::ChR2), p<0.0001; $F_{(4,50)}$=44.80 (opto-β2AR), p<0.0001; $F_{(1,20)}$=0.342 (opto-β2AR+U69593), p=0.5651; Cond=conditioning

Supplementary Table 6: CPA score (mean±SEM) and statistical analysis for raw CPA score represented in Figure 6h and Supplementary Figure 7h respectively.

	Astrocytic β2AR				Neuronal β2AR			
	Cond-NC-β2AR		Cond-knockdown-GFAP::β2AR		Cond-NC-β2AR		Cond-knockdown-hSyn::β2AR	
Test days	Mean	SEM	Mean	SEM	Mean	SEM	Mean	SEM
1	316.50	7.95	120.83	7.45	311.16	11.61	296.66	13.28
4	273.16	10.79	116.16	8.17	277.83	8.84	287.66	7.50
7	195.33	10.72	111.83	7.16	226.50	16.14	211.16	17.80
10	117.66	7.72	73.50	7.86	120.50	8.25	129.50	11.31
14	28.83	6.08	25.50	5.03	34.50	3.49	35	5.02

Statistics: $F_{(4,50)}$=48.06 (astrocytic β2AR knockdown), p<0.0001; $F_{(4,50)}$=0.5978 (neuronal β2AR knockdown), p=0.6659; Cond=conditioning

Supplementary Table 7: CPA score (mean±SEM) and statistical analysis for raw CPA score represented in Figure Supplementary Figure 9d, e and Figure 7d respectively.

	Training				Testing							
	Cond-saline		Cond-CNO		Cond-saline		Cond-CNO		Cond.ChR2+sal-ACC		Cond.ChR2+CNO-ACC	
Test days	Mean	SEM	Mean	SEM	Mean	SEM	Mean	SEM	Mean	SEM	Mean	SEM
1	317.66	13.3	110.33	5.14	298.66	6.29	117	6.61	414.83	11.11	97.66	11.20
4	251.83	19.3	68.16	5.20	218.83	7.91	84	7.82	376	12.42	95.1	8.24
7	179.50	10.5	52	5.48	150.83	10.95	47.5	5.51	355.50	10.95	87.83	9.45
10	66.50	5.76	25.66	3.99	53.83	8.63	27.75	4.38	338.50	8.37	71	7.37
14	17.50	3.99	15	2.14	16.52	2.94	23.75	5.17	302.16	8.91	53	9.33

Statistics: $F_{(4,50)}$=48.24 (training), p<0.0001; $F_{(4,50)}$=77.09 (testing), p<0.0001; $F_{(4,50)}$=3.32 (ChR2-CNO-ACC), p=0.017; Cond=conditioning
