# Supplementary material for: Adrenergic signalling to astrocytes in anterior cingulate cortex contributes to pain-related aversive memory in rats
Source: Commun Biol. 2023 Jan 5;6:10. doi: 10.1038/s42003-022-04405-6 (PMC9816175; doi:10.1038/s42003-022-04405-6)
Supplement: Supplementary file 2 — Description of additional supplementary files [file 42003_2022_4405_MOESM2_ESM.pdf]

Description of Additional Supplementary Files

File name: Supplementary Data 1
Description: The source data behind the Figures presented in main text and supplementary.

File name: Supplementary Data 2
Description: Uncropped and unedited blot images behind the Figures 3c-h, 5g-I, 6g, 7g-I, and Supp Figure 5e, 7g, 9f-k.
