# Supplementary material for: Adrenergic signalling to astrocytes in anterior cingulate cortex contributes to pain-related aversive memory in rats
Source: Commun Biol. 2023 Jan 5;6:10. doi: 10.1038/s42003-022-04405-6 (PMC9816175; doi:10.1038/s42003-022-04405-6)
Supplement: Supplementary file 5 — Reporting summary [file 42003_2022_4405_MOESM5_ESM.pdf]

nature portfolio

Corresponding author(s): Ying Li

Last updated by author(s): Dec 8, 2022

Reporting Summary

Nature Portfolio wishes to improve the reproducibility of the work that we publish. This form provides structure for consistency and transparency in reporting. For further information on Nature Portfolio policies, see our Editorial Policies and the Editorial Policy Checklist.

Statistics

For all statistical analyses, confirm that the following items are present in the figure legend, table legend, main text, or Methods section.

n/a	Confirmed	
☐	☒	The exact sample size (*n*) for each experimental group/condition, given as a discrete number and unit of measurement
☐	☒	A statement on whether measurements were taken from distinct samples or whether the same sample was measured repeatedly
☐	☒	The statistical test(s) used AND whether they are one- or two-sided<br>*Only common tests should be described solely by name; describe more complex techniques in the Methods section.*
☒	☐	A description of all covariates tested
☐	☒	A description of any assumptions or corrections, such as tests of normality and adjustment for multiple comparisons
☐	☒	A full description of the statistical parameters including central tendency (e.g. means) or other basic estimates (e.g. regression coefficient) AND variation (e.g. standard deviation) or associated estimates of uncertainty (e.g. confidence intervals)
☐	☒	For null hypothesis testing, the test statistic (e.g. F, t, r) with confidence intervals, effect sizes, degrees of freedom and P value noted<br>*Give P values as exact values whenever suitable.*
☒	☐	For Bayesian analysis, information on the choice of priors and Markov chain Monte Carlo settings
☒	☐	For hierarchical and complex designs, identification of the appropriate level for tests and full reporting of outcomes
☒	☐	Estimates of effect sizes (e.g. Cohen's *d*, Pearson's *r*), indicating how they were calculated

Our web collection on statistics for biologists contains articles on many of the points above.

Software and code

Policy information about availability of computer code

Data collection	This study did not generate code.
Data analysis	This study did not generate code.

For manuscripts utilizing custom algorithms or software that are central to the research but not yet described in published literature, software must be made available to editors and reviewers. We strongly encourage code deposition in a community repository (e.g. GitHub). See the Nature Portfolio guidelines for submitting code & software for further information.

Data

Policy information about availability of data

All manuscripts must include a data availability statement. This statement should provide the following information, where applicable:

- Accession codes, unique identifiers, or web links for publicly available datasets
- A description of any restrictions on data availability
- For clinical datasets or third party data, please ensure that the statement adheres to our policy

The data supporting the current study have not been deposited to any public repository but are available from the corresponding author on request.

Field-specific reporting

Please select the one below that is the best fit for your research. If you are not sure, read the appropriate sections before making your selection.

☒ Life sciences ☐ Behavioural & social sciences ☐ Ecological, evolutionary & environmental sciences

For a reference copy of the document with all sections, see nature.com/documents/nr-reporting-summary-flat.pdf

Life sciences study design

All studies must disclose on these points even when the disclosure is negative.

Sample size	We used power analysis to estimate the sample size. For western blot studies, power calculation of one-way ANOVA predicted that four or five rats per group is necessary to gain the power of 0.8 and error probability of 0.05. A similar power analysis was calculated for behavioural experiments and indicated a minimum sample size requirement of six animals in each study for two-way ANOVA to achieve a power of 0.8 and an error probability of 0.05.
Data exclusions	No data was excluded.
Replication	The reproducibility of the experimental findings were confirmed.
Randomization	Adult male Sprague Dawley (SD) rats (250-300 grams) were used in the current study and they were randomly allocated to different experiments.
Blinding	The investigators were blinded to group allocation during data analysis.

Reporting for specific materials, systems and methods

We require information from authors about some types of materials, experimental systems and methods used in many studies. Here, indicate whether each material, system or method listed is relevant to your study. If you are not sure if a list item applies to your research, read the appropriate section before selecting a response.

Materials & experimental systems

n/a	Involved in the study	
☐	☒	Antibodies
☒	☐	Eukaryotic cell lines
☒	☐	Palaeontology and archaeology
☐	☒	Animals and other organisms
☒	☐	Human research participants
☒	☐	Clinical data
☒	☐	Dual use research of concern

Methods

n/a	Involved in the study	
☒	☐	ChIP-seq
☒	☐	Flow cytometry
☒	☐	MRI-based neuroimaging

Antibodies

Antibodies used	Rabbit anti-DβH Abcam Cat. No. ab96615; Biotinylated goat anti-rabbit IgG secondary antibody Vector Laboratories Cat. No. BA-1000; Mouse anti-GFAP Sigma-Aldrich Cat. No. G3893; Rabbit anti-GFAP Abcam Cat. No. ab7260; Mouse anti-NeuN Millipore Cat. No. MAB-377; Mouse anti-tyrosine hydroxylase Millipore Cat. No. MAB-318; Mouse anti-β2AR Santa Cruz Cat. No. sc271322; Rabbit anti-β2AR Thermo Scientific Cat. No. PA5 86339; Rabbit anti- β1AR Almone Labs Cat. No. AAR-023; Rabbit anti-S100β Abcam Cat. No. ab41548; Rabbit anti-c-Fos Synaptic Systems Cat. No. 226017; Rabbit anti-pCREB Millipore Cat. No. 06-519; Rabbit anti-CREB Millipore Cat. No.04-767; Rabbit anti-ERK1/2 Cell Signaling Technology Cat. No. 197G2; Mouse anti-α tubulin Sigma Cat. No. T6074; rabbit anti-Iba1 Abcam Cat. No. 178846; mouse anti-Iba1 Abcam Cat. No. 283319; chicken anti-GFP Abcam Cat. No. ab13970.
Validation	The relevant antibodies were validated by the manufacturers.

Animals and other organisms

Policy information about studies involving animals; ARRIVE guidelines recommended for reporting animal research

Laboratory animals	All the experimental work was carried out on adult male Sprague Dawley (SD) rats weighing about 250-300 grams.
Wild animals	No wild animals were used in this study.
Field-collected samples	No field-collected samples were used in this study.

Ethics oversight	Animal studies were performed in accordance with the guidelines laid down by the Committee on the Use and Care of Animals, Department of Health, Govt. of Hong Kong SAR [Animals (Control of Experiments) Ordinance (Cap. 340), License to Conduct Experiments Ref: (19-155) in DH/HT&A/8/2/5 Pt. 1, (19-157) in DH/HT&A/8/2/5 Pt. 1 and (20-16) in DH/HT & A/8/2/5 Pt. 1]. Approvals for “Ethical Review of Research Experiments involving Animal Subjects” were granted by Animal Research Ethics Sub-Committee, City University of Hong Kong (Ref: A-0557).

Note that full information on the approval of the study protocol must also be provided in the manuscript.
